# Clinical Significance of a Novel Tumor Progression-Associated Immune Signature in Colorectal Adenocarcinoma

**DOI:** 10.3389/fcell.2021.625212

**Published:** 2021-02-25

**Authors:** Rui Mao, Fan Yang, Zheng Wang, Chenxin Xu, Qian Liu, Yanjun Liu, Tongtong Zhang

**Affiliations:** ^1^The Center of Gastrointestinal and Minimally Invasive Surgery, Affiliated Hospital of Southwest Jiaotong University, Chengdu, China; ^2^Emergency Department, Peking University Third Hospital, School of Medicine, Peking University, Beijing, China; ^3^Department of Colorectal Surgery, National Cancer Center, National Clinical Research Center for Cancer, Cancer Hospital, Chinese Academy of Medical Sciences and Peking Union Medical College, Beijing, China; ^4^The Center of Gastrointestinal and Minimally Invasive Surgery, Department of General Surgery, The Third People’s Hospital of Chengdu, Chengdu, China; ^5^Medical Research Center, The Third People’s Hospital of Chengdu, The Affiliated Hospital of Southwest Jiaotong University, The Second Chengdu Hospital Affiliated to Chongqing Medical University, Chengdu, China

**Keywords:** adenocarcinoma, immune signature, colorectal prognosis, qRT-PCR, recurrence

## Abstract

**Background:**

Some colorectal adenocarcinoma (CRC) patients are susceptible to recurrence, and they rapidly progress to advanced cancer stages and have a poor prognosis. There is an urgent need for efficient screening criteria to identify patients who tend to relapse in order to treat them earlier and more systematically.

**Methods:**

We identified two groups of patients with significantly different outcomes by unsupervised cluster analysis of GSE39582 based on 101 significantly differentially expressed immune genes. To develop an accurate and specific signature based on immune-related genes to predict the recurrence of CRC, a multivariate Cox risk regression model was constructed with a training cohort composed of 519 CRC samples. The model was then validated using 129, 292, and 446 samples in the real-time quantitative reverse transcription PCR (qRT-PCR), test, and validation cohorts, respectively.

**Results:**

This classification system can also be used to predict the prognosis in clinical subgroups and patients with different mutation states. Four independent datasets, including qRT-PCR and The Cancer Genome Atlas (TCGA), demonstrated that they can also be used to accurately predict the overall survival of CRC patients. Further analysis suggested that high-risk patients were characterized by worse effects of chemotherapy and immunotherapy, as well as lower immune scores. Ultimately, the signature was identified as an independent prognostic factor.

**Conclusion:**

The signature can accurately predict recurrence and overall survival in patients with CRC and may serve as a powerful prognostic tool to further optimize cancer immunotherapy.

## Introduction

Colorectal adenocarcinoma is the second most common cancer in men and the third most common cancer in women worldwide (Thrumurthy et al., 2016; Miller et al., 2019). The 5-year relative survival rate of CRC patients is 65%. For patients diagnosed with stage I and stage II CRC, the 5-year relative survival rates are 91 and 82%, respectively. However, the 5-year survival rate of stage IV disease is only 12% (Miller et al., 2019; Siegel et al., 2020). Additionally, even in early-stage patients, there is a significant recurrence rate after surgical removal, and patients with relapse tend to have a poor prognosis (Thrumurthy et al., 2016; Lefèvre et al., 2019). Novel molecular biomarkers that can precisely indicate the stage of disease progression and predict clinical outcomes are urgently needed.

Immunotherapy has been investigated in multiple solid tumors, including CRC (Siegel et al., 2020). Increased expressions of inhibitory checkpoint proteins [including programmed cell death 1 (PD1), PD1 ligand 1 (PDL1), PDL2, and cytotoxic T lymphocyte protein 4 (CTLA4)] are common features of CRC that protect tumors from destruction by T cells (Juneja et al., 2017). Immune checkpoint therapy targets these common suppressive signals expressed by T cells and tumor cells to enhance antitumor immune responses (Curran et al., 2010; Pentcheva-Hoang et al., 2014). In addition to checkpoint proteins, coinhibitory ligands such as B7-H3 (also known as CD276) and B7X (also known as B7-H4) exhibit upregulated expression in many solid tumors, including CRC. Other potential immune checkpoint proteins, such as HHLA2 (Janakiram et al., 2015a), TMIGD2, TIM3 (Romero, 2016), and LAG3, are also overexpressed in some tumors, where they mediate immune suppression (Xiao and Freeman, 2015; Koyama et al., 2016).

The immune response in the tumor microenvironment is considered to be an important factor in determining tumor aggressiveness, progression, and response to immunomodulators. The density and type of tumor-infiltrating immune cells, as well as their cytokine and immune-related gene (IRG) expression, have been extensively studied as prognostic biomarkers for CRC (Koch et al., 2006; Dupaul-Chicoine et al., 2015). In addition, previous studies have reported the important value of using genes or lncRNA signatures to predict recurrence and prognosis in patients with CRC (Tian et al., 2017; Mu et al., 2020). However, whether these IRG signatures can also be used as predictors of recurrence and prognosis of CRC remains to be explored.

Considering the increasing proportion of patients diagnosed with early-stage CRC and the poor prognosis of advanced-stage patients, there is an urgent need to develop an IRG-based recurrence signature (IGBRS) in patients with CRC. Comprehensive analysis of IRG and the tumor microenvironment in CRC can improve the stratification of the risk of CRC patients clinically and allow exploration of possible biotherapeutic targets. In the current study, we integrated 1,386 CRC cases with recurrence-free survival (RFS) data and 1,375 cases with OS data from eight independent cohorts; a dataset from TCGA; and GSE14333, GSE17538, GSE33113, GSE37892, GSE38832, GSE39582, and real-time quantitative reverse transcription PCR (qRT-PCR) cohorts to establish a novel robust IGBRS. Further, we studied whether IGBRS can predict clinical outcomes independent of the clinical and pathological characteristics and molecular subtype. We also explored the differences in the immune landscape and somatic mutation pattern between high- and low-risk groups.

## Materials and Methods

### Data Acquisition and Preprocessing

**FIGURE 1:**
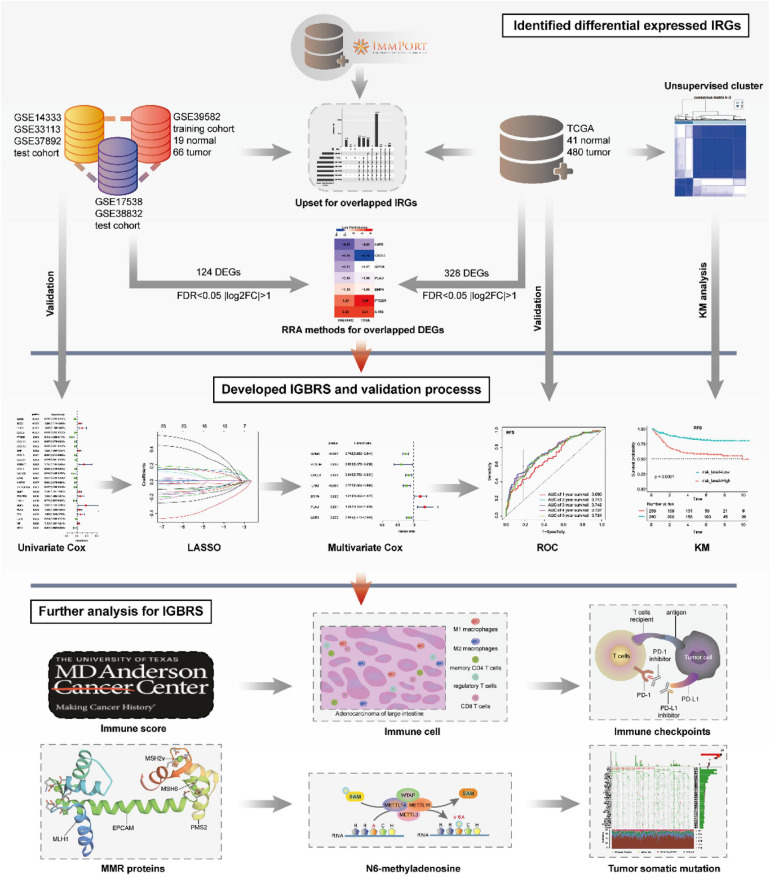
The entire analytical process of the study.

The entire analytical process of the study is presented in Figure 1.

The GSE14333, GSE17538, GSE33113, GSE37892, GSE38832, GSE35640, GSE63557, and GSE39582 datasets were downloaded from the GEO^1^ database with log2 transformation and quantile-normalized matrices. In general, the expression of genes with multiple probes is based on the median. The mRNA expression profiles were in the fragments per kilobase of transcript per million mapped reads (FPKM) format. The data used in this study met the following criteria: (1) the expression level of each probe must be greater than 0 in ≥75% of the samples and (2) data with respect to survival time and survival status must be available for each patient. GSE39582 was developed as a training cohort including 519 patients with RFS and 562 patients with OS. We combined the GSE17538 and GSE38832 datasets as a test cohort because they collectively have RFS (292 patients) and disease-specific survival (DSS, 299 patients) data. In addition, the GSE14333, GSE33113, and GSE37892 datasets were combined as a validation cohort owing to their single RFS (446 patients) data. When combining the GSE datasets, we used the combat function of the R software package sva to remove the batch effect. Additionally, GSE17538 contained 232 patients with OS data.

Moreover, between July 2013 and August 2015, a total of 129 frozen surgically resected tumor tissues were obtained from patients with a pathological diagnosis of CRC at the Department of Colorectal Surgery, National Cancer Center/National Clinical Research Center for Cancer/Cancer Hospital, Chinese Academy of Medical Sciences and Peking Union Medical College.

The TCGA-COAD and TCGA-SKCM datasets were downloaded from TCGA^2^, including mRNA expression profiles of 452 CRC specimens and the corresponding clinical follow-up data. The basic information of the dataset included in this study is shown in Supplementary Table 1.

### Identification of Differentially Expressed IRGs in CRC and Adjacent Normal Tissues

We used the IRGs obtained from the ImmPort database^3^ to intersect with the mRNA data in the TCGA and GEO database matrices. Then, the edgeR package in R software (Robinson et al., 2010) was used to analyze the difference in expression between CRC and adjacent normal tissues of the TCGA and GSE39582 datasets of common IRGs. Significantly differentially expressed RNAs were identified by setting adjusted *P*-values < 0.05 and | log_2_[fold change (FC)]| > 1. We chose the IRGs that were significantly differentially expressed in both databases at the same time. We plotted a volcano map using the ggplot2 package.

### Unsupervised Clustering for Differentially Expressed IRGs

Unsupervised cluster analysis was used to classify the patients according to the expression of 101 differentially expressed IRGs identified by differential analysis. The number of clusters and their stability were determined by the consensus clustering algorithm (McLachlan et al., 2017). We performed these steps based on the ConsensusClusterPlus package (Wilkerson and Hayes, 2010) and repeated it 1,000 times to ensure the stability of the classification.

### Prognostic Evaluation Using the IGBRS

The prognostic value of each IRG was first calculated by univariate Cox analysis with the R/survival package, and IRGs with *P* < 0.05 were selected as seed IRGs for Cox LASSO regression. Next, multivariate Cox regression was applied to identify prognostic signatures with the R packages glment, survminer, and survival. The risk scores for each patient in the training group were calculated based on the following formula: risk score = Σ{expGene(*n*) × βGene(*n*)}, where exp is the prognostic gene expression level and β is the multivariate Cox regression model regression coefficient (Yang et al., 2019). All samples were randomly divided into high- and low-risk score sets, with the median risk score as the cutoff value. The Kaplan–Meier method was used to generate survival curves for each group, and the log-rank test was used to determine the statistical significance of differences. The R packages survivalROC and timeROC were used to plot and visualize ROC curves to calculate the AUC and confidence intervals (CIs) to evaluate the diagnostic accuracy of the IGBRS.

### Real-Time Quantitative Reverse Transcription PCR

RNA was extracted from the frozen samples using TRIzol reagent (Takara, #9109) according to standard protocols. Total RNA was reverse transcribed into cDNA with random primers using the Transcriptor First Strand cDNA Synthesis Kit (Roche, Penzberg, Germany) following the manufacturer’s instructions. RNA expression levels were measured by real-time quantitative reverse transcription PCR (qRT-PCR) using FastStart Essential DNA Green Master mix (Roche, Penzberg, Germany) on a Roche LightCycler 480 (Roche, Penzberg, Germany) with the following PCR conditions: pre-denaturation at 95°C for 2 min, followed by 45 cycles of denaturation at 94°C for 15 s, annealing at 55°C for 15 s, and extension at 68°C for 30 s. The melting curve was prepared at 65–95°C. The mRNA expression of each of the seven genes was normalized to GAPDH levels. All quantitative PCR analyses were conducted in triplicate and the average value was calculated. Relative gene expression was analyzed by the 2^–Δ^ ^Δ^ ^*Ct*^ method. We verified the specificity of the PCR primers using BLAST. A single peak in the melting curve indicated that the PCR products were specific. The primers used in the study are presented in Supplementary Table 2.

### Validation of the Prognostic Value of the IGBRS

The IGBRS was validated in different clinical subgroups and histopathological subtypes. A similar analysis process was also applied to the qRT-PCR, test, validation, and TCGA cohorts. Univariate and multivariate Cox regression analyses of the IGBRS and other clinicopathological factors were performed to evaluate whether the IGBRS is an independent risk factor for CRC.

### Exploration of the Relationships Between the IGBRS and Immunity

The ESTIMATE algorithm was used to determine the stromal score, the ESTIMATE score, and immune scores of each sample with R software, and the differences in the degree of immune cell infiltration between the high- and low-risk groups were further compared using the Wilcoxon test (Runa et al., 2017). The CIBERSORT.R package was used to assess the proportions of 22 immune cell subtypes based on the expression profile (Newman et al., 2015). CRC samples with *P* < 0.05 in the CIBERSORT analysis results were used in further analyses. The Mann–Whitney *U* test was used to compare differences in immune cell subtypes in the high-risk and low-risk groups. We also compared the differences in the expression of 49 immune checkpoints {including the B7-CD28 family [*CD274* (*PD-L1*), *B7-H3*, *CTLA4*, *ICOSLG*, *PD-L2*, *TMIGD2*, *ICOS*, *PD-1*, and *HHLA2*] (Janakiram et al., 2015b; Zhang et al., 2018), the TNF superfamily [*TNFRSF14*, *TNFRSF18*, *TNFRSF25*, *TNFRSF4*, *TNFRSF8*, *TNFRSF9*, *TNFSF14*, *TNFSF15*, *TNFSF18*, *TNFSF4*, *TNFSF9*, *CD40*, *BTLA*, *CD27*, *CD40LG*, and *CD70*] (Ward-Kavanagh et al., 2016), and several other immune checkpoint members [*ADORA2A*, *BTNL2*, *CD160*, *CD200*, *CD200R1*, *CD244*, *CD28*, *CD44*, *CD48*, *CD80*, *CD86*, *ENTPD1*, *FGL1*, *HAVCR2*, *IDO1*, *IDO2*, *KIR3DL1*, *LAG3*, *LAIR1*, *LGALS9*, *NRP1*, *NCR3*, and *TIGIT*] (Chrétien et al., 2019; Wang et al., 2019a, b)} between the high- and low-risk groups to analyze the landscape of genetic variation (R package maftools). Moreover, we calculated the Pearson correlation coefficients between seven IRGs and differentially expressed immune checkpoint genes based on the gene expression. In addition, we calculated the Pearson correlation coefficients between seven IRGs and immune cells based on the gene expression of these IRGs and immune cell infiltration in each sample. *P* < 0.05 was considered to be statistically significant. We corrected *P*-values by the Benjamini–Hochberg method (Hochberg and Benjamini, 1990).

### Gene Set Enrichment Analysis

Gene set enrichment analysis (GSEA) was performed using the software GSEA v4.0.3^4^. We divided all samples into high- and low-risk score groups according to the median cutoff value of the risk score. We input the profiles of the adjusted expression data for all transcripts, groups of high- and low-risk score samples, and gene set files (c2. cp. kegg. v6. 1. symbols. gmt). Enrichment *P*-values were based on 10,000 permutations and were subsequently adjusted for multiple testing using the Benjamini–Hochberg procedure to control the false discovery rate (FDR) (Hochberg and Benjamini, 1990).

### Tumor Somatic Mutation Analysis

The waterfall function of the maftools package was used to present the mutation landscapes in patients with high- and low-risk score subtypes in the TCGA-COAD cohort. The total number of somatic mutations was adopted to assess the mutation burden, which is convenient and significantly correlated with the number of non-synonymous mutations. Missense, nonsense, non-stop, silent, and frameshift/in-frame insertions and deletions were counted and summed, and germline mutations without somatic mutations were excluded (Shen et al., 2018). According to the median tumor mutation burden (TMB), all CRC samples with somatic mutations in the TCGA dataset were divided into the high-TMB group and the low-TMB group.

### Prognostic Meta-Analysis

In order to clarify the comprehensive prognostic value of IGBRS in four different groups, prognostic meta-analysis was carried out using STATA software (version 12.0). Then, the random effect model was used to calculate the combined hazard ratio (HR) value.

## Results

### Identification of Differentially Expressed IRGs and Unsupervised Cluster Analysis

**FIGURE 2:**
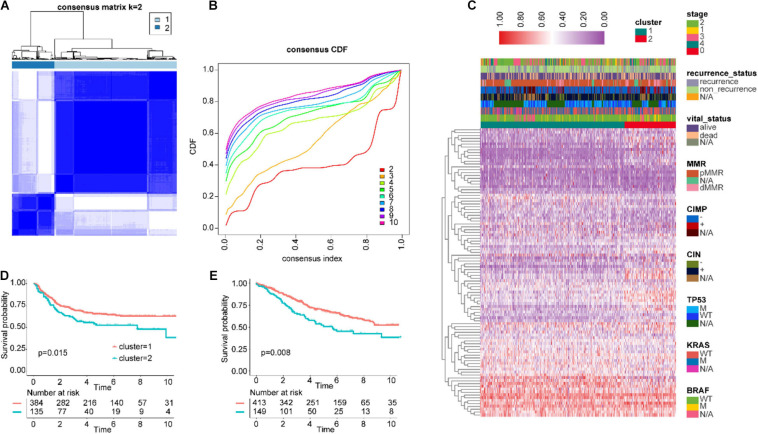
Unsupervised clustering for differentially expressed IRGs. **(A,B)** Classification of GSE39582 into two groups. **(C)** Landscape of the expression of 101 IRGs in the GSE39582 set. **(D)** Kaplan–Meier curves of RFS in the training cohort based on clustering. **(E)** Kaplan–Meier curves of OS in the training cohort based on clustering. IRGs, immune-related genes; RFS, recurrence-free survival; OS, overall survival.

First, we combined the two datasets GSE17538 and GSE38832 as the test cohort and merged GSE14333, GSE33113, and GSE37892 as the validation cohort and removed the batch effect (Supplementary Figures 1A,B). Then, IRGs coincident in the TCGA, GSE39582, test, validation, and ImmPort databases were screened (Supplementary Figure 1C), and difference analysis was performed in CRC and adjacent normal tissues of TCGA and GSE39582. Next, we obtained 328 and 124 differentially expressed IRGs from the two respective datasets. Ultimately, we identified a total of 101 overlapping IRGs from two cohorts (Supplementary Figure 1D). The log_2_(FC) values of seven IRGs in the IGBRS are presented in Supplementary Figure 1E. Based on 101 differentially expressed IRGs, GSE39582 was divided into two groups by unsupervised clustering (Figures 2A–C). The prognostic analysis of the two groups showed that cluster-1 had significant advantages in survival and no recurrence (Figures 2D,E).

### Construction of the IGBRS for CRC With the Training Cohort

**FIGURE 3:**
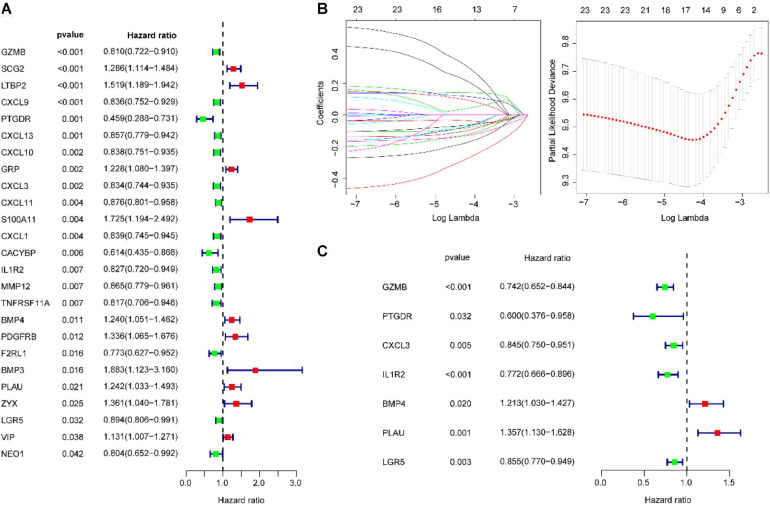
Identification of prognostic IRGs in CRC. **(A)** Univariate Cox regression analysis identified 25 IRGs significantly associated with OS. **(B)** LASSO coefficient profiles of the 25 IRGs. A coefficient profile plot was produced against the log_2_(lambda) sequence. A vertical line was drawn at the value selected using 100-fold cross-validation, where optimal lambda resulted in seven IRGs with non-zero coefficients. **(C)** Multivariate Cox regression analysis confirmed seven independent molecules affecting the prognosis of patients with CRC. IRG, immune-related gene; CRC, colorectal adenocarcinoma.

A total of 101 IRGs were evaluated by univariate Cox survival analysis, and 25 IRGs with *P* < 0.05 were filtered out and included in subsequent analyses (Figure 3A). As shown in Figure 3B, LASSO regression analysis identified seven IRGs (based on lambda.lse criteria) that were subjected to multivariate Cox regression analysis (Figure 3C). The results are presented in Supplementary Table 3. Ultimately, we identified seven IRGs predicting CRC patient recurrence, namely, BMP4, CXCL3, GZMB, IL1R2, LGR5, PLAU, and PTGDR. The risk score of the IGBRS was calculated based on the following formula: risk score = (0.1927 × expression of *BMP4*) − (0.1689 × expression of *CXCL3*) − (0.2983 × expression of *GZMB*) − (0.1572 × expression of *LGR5*) − (0.0968 × expression of *IL1R2*) − (0.5107 × expression of *PTGDR*) + (0.3049 × expression of *PLAU*). The expression levels of the seven IRGs in the training (panel A), validation (panel B), test (panel C), and TCGA (panel D) cohorts are shown in Supplementary Figure 2.

**FIGURE 4:**
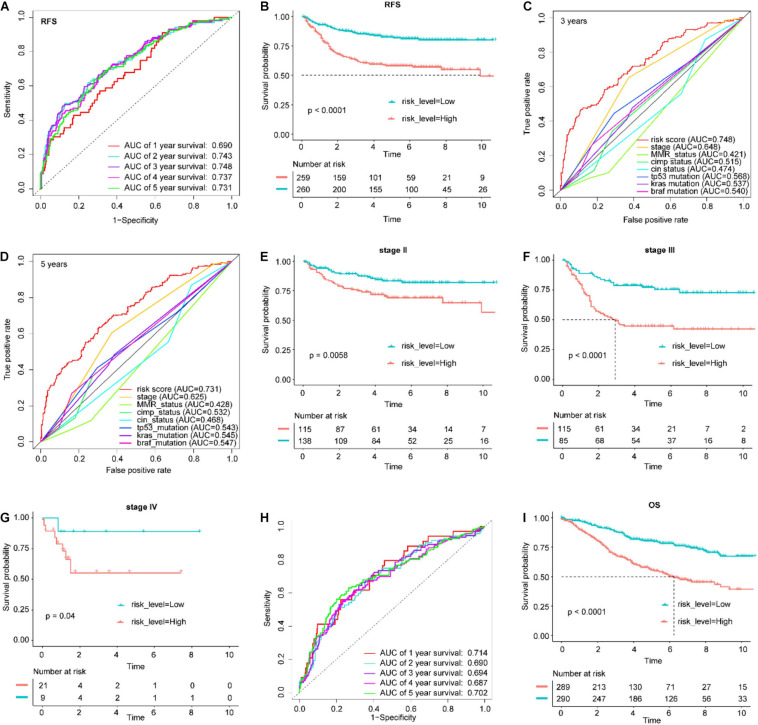
Application of the IGBRS in predicting RFS and OS of CRC patients in the training cohort. **(A)** ROC analysis of the IRG signature for the prediction of 1-, 2-, 3-, 4-, and 5-year RFS in the training cohort. **(B)** Kaplan–Meier curves of RFS in all CRC patients of the training cohort based on the risk score. **(C)** ROC analysis of the IRG signature and the TNM stage for the prediction of 3-year RFS in the training cohort. **(D)** ROC analysis of the IRG signature and the TNM stage for the prediction of 5-year RFS in the training cohort. **(E)** Kaplan–Meier curves of RFS in stage II CRC patients of the training cohort based on the risk score. **(F)** Kaplan–Meier curves of RFS in stage III CRC patients of the training cohort based on the risk score. **(G)** Kaplan–Meier curves of RFS in stage IV CRC patients of the training cohort based on the risk score. **(H)** ROC analysis of the IRG signature for the prediction of 1-, 2-, 3-, 4-, and 5-year OS in the training cohort. **(I)** Kaplan–Meier curves of OS in all CRC patients of the training cohort based on the risk score. IRG, immune-related gene; CRC, colorectal adenocarcinoma; RFS, recurrence-free survival; OS, overall survival; IGBRS, IRG-based recurrence signature; ROC, receiver operating characteristic.

The patients in the training set were divided into a high-risk group and a low-risk group, with the median risk score as the cutoff point (Supplementary Figure 3A). The patients in the high-risk group had significantly worse RFS than those in the low-risk group (log-rank test *P*-value < 0.0001, Figure 4B). In addition, ROC analysis was implemented to determine whether survival predictions made with the IGBRS were accurate (Figure 4A). The AUC values were assessed for 3-year (AUC = 0.748) and 5-year (AUC = 0.731) RFS, and these values were higher than the AUC of the traditional TNM pathological staging system (Figures 4C,D). The results suggest that the IGBRS can be used to effectively evaluate the recurrence of CRC patients. Furthermore, patients in the high-risk group had worse RFS than those in the low-risk group for pathological stage II (*P* = 0.0058), pathological stage III (*P* < 0.0001), and pathological stage IV (*P* = 0.04) (Figures 4E–G), which means that the IGBRS can create a good risk stratification in different pathological stages of CRC. Similarly, to explore whether the IGBRS is equally valid for predicting the OS of CRC patients, we conducted a similar analysis process on the patients with OS data. Comparisons of OS showed that the mortality rate in the high-risk group was significantly higher than that in the low-risk group (Figure 4I, *P* < 0.0001). The AUC for 5-year OS using the predictive nomogram reached 0.702 (Figure 4H).

### Verification of the Prognostic Classifier With the qRT-PCR Cohort

**FIGURE 5:**
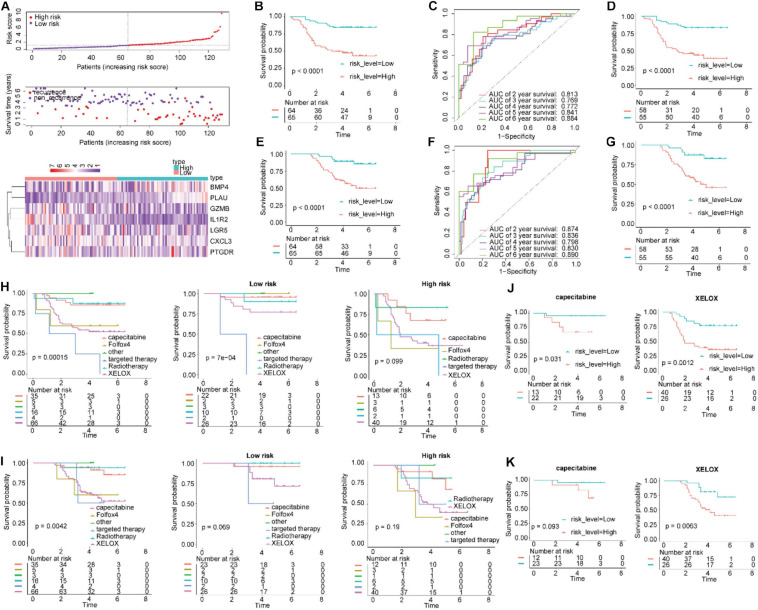
Application of the IGBRS in predicting RFS and OS of CRC patients in the qRT-PCR cohort. **(A)** The distribution of the risk score, recurrence status, and gene expression panel in the qRT-PCR cohort. **(B)** Kaplan–Meier curves of RFS in all CRC patients of the qRT-PCR cohort based on the risk score. **(C)** ROC analysis of the IRG signature for the prediction of 2-, 3-, 4-, 5-, and 6-year RFS in the qRT-PCR cohort. **(D)** Kaplan–Meier curves of RFS in CRC patients with chemotherapy based on the risk score in the qRT-PCR cohort. **(E)** Kaplan–Meier curves of OS in all CRC patients of the qRT-PCR cohort based on the risk score. **(F)** ROC analysis of the IRG signature for the prediction of 2-, 3-, 4-, 5-, and 6-year OS in the qRT-PCR cohort. **(G)** Kaplan–Meier curves of OS in CRC patients with chemotherapy based on the risk score in the qRT-PCR cohort. **(H)** Kaplan–Meier curves of RFS in CRC patients based on various treatments in the qRT-PCR cohort, high-risk group, and low-risk group. **(I)** Kaplan–Meier curves of OS in CRC patients based on various treatments in the qRT-PCR cohort, high-risk group, and low-risk group. **(J)** Kaplan–Meier curves of RFS in CRC patients treated with capecitabine and XELOX in the qRT-PCR cohort. **(K)** Kaplan–Meier curves of OS in CRC patients treated with capecitabine and XELOX in the qRT-PCR cohort. IRG, immune-related gene; CRC, colorectal adenocarcinoma; RFS, recurrence-free survival; OS, overall survival; IGBRS, IRG-based recurrence signature; ROC, receiver operating characteristic; qRT-PCR, real-time quantitative reverse transcription PCR.

To determine the robustness of this signature, this verification process was also performed for the qRT-PCR cohort. By using the same risk score formula in the validation set, we classified patients into high-risk score (*n* = 64) and low-risk score (*n* = 65) groups, with the median score as the cutoff point (Figure 5A). The results showed that the Kaplan–Meier curves for RFS suggested that the patients with high-risk scores had significantly worse RFS than those with low-risk scores (log-rank test *P*-value < 0.0001, Figure 5B). The AUC for 6-year RFS reached 0.884 (Figure 5C). We conducted a similar analysis process on the patients with OS data. Comparisons of OS showed that the mortality rate in the high-risk group was significantly higher than that in the low-risk group (Figure 5E, *P* < 0.0001). The AUC for 6-year OS using the IGBRS reached 0.890 (Figure 5F).

Additionally, in all CRC patients who underwent chemotherapy, the patients in the high-risk groups had shorter RFS and OS times than those in the low-risk groups (Figures 5D,G; *P* < 0.0001). These results indicate that the IGBRS-based risk score is a promising prognosis predictor whether or not the alluded chemotherapy drugs were administered (Figures 5H–K).

### Verification of the Prognostic Classifier With the Test Cohort

In agreement with the abovementioned findings, we also validated the robustness of the IGBRS with the test dataset (*n* = 292). We classified the patients into high-risk score (*n* = 146) and low-risk score (*n* = 146) groups, with the median score as the cutoff point (Supplementary Figure 3B). The AUC for 3-year recurrence prediction by the IGBRS reached 0.754 (Supplementary Figure 4A). The Kaplan–Meier curves suggested that the patients with high-risk scores had significantly worse RFS than those with low-risk scores (log-rank test *P*-value < 0.0001, Supplementary Figure 4B). Furthermore, patients in the high-risk group had worse RFS than those in the low-risk group for pathological stage II (*P* = 0.0012), pathological stage III (*P* = 0.039), and pathological stage IV (*P* = 0.025) (Supplementary Figures 4C–E), which demonstrated that the IGBRS can create a good risk stratification in different pathological stages of CRC. To explore whether the IGBRS is equally valid for predicting the DSS of CRC patients, we conducted a similar analysis process on the patients with DSS data. The AUC for 3-year DSS using the predictive nomogram reached 0.752 (Supplementary Figure 4F). Comparisons of DSS showed that the patients in the high-risk group had a significantly higher mortality rate than those in the low-risk group (Supplementary Figure 4G, *P* < 0.0001). Furthermore, we validated the ability of the IGBRS to predict OS for the 232 patients with OS data in the GSE17538 set. The results show that the AUC for 3-year OS reached 0.721 (Supplementary Figure 4H), and the patients with a high-risk score had a significantly higher mortality rate than those with a low-risk score (Supplementary Figure 4I, *P* < 0.0001).

### Verification of the Prognostic Classifier With the Validation and TCGA Cohorts

To validate the robustness of the IGBRS, this verification process was also performed for the validation and TCGA cohorts. Using the same risk score formula in the validation set, we classified patients into high-risk score (*n* = 223) and low-risk score (*n* = 223) groups, with the median score as the cutoff point (Supplementary Figure 5A). The results showed that the AUC for 5-year RFS reached 0.660 (Supplementary Figure 5B). The Kaplan–Meier curves for RFS suggested that the patients with high-risk scores had significantly worse RFS than those with low-risk scores (log-rank test *P*-value = 0.00015, Supplementary Figure 5C). In addition, we observed that the AUC values for the assessment of 5-year RFS (AUC = 0.624) by IGBRS were higher than those of the Duke staging system (AUC = 0.487) in the GSE14333 set (Supplementary Figure 5D). In the GSE33113 and GSE37892 cohorts, the AUC values for the assessment of 5-year RFS (AUC = 0.700) by the IGBRS were higher than those of the traditional TNM pathological staging system (AUC = 0.673) (Supplementary Figure 5E). It is important that, in the pathological stage II and III subgroups, the patients in the high-risk group had worse RFS than those in the low-risk group (Supplementary Figures 5F,G; *P* < 0.05).

Using the same signature score formula in the TCGA set (*n* = 452), we classified the patients into groups with a high-risk score (*n* = 226) and a low-risk score (*n* = 226) by taking the median score as the cutoff point (Supplementary Figure 6A). The AUC exhibited by the IGBRS for 5*-*year survival reached 0.686 (Supplementary Figure 6B). The Kaplan*–*Meier OS curves suggested that patients with high-risk scores had significantly worse OS than those with low-risk scores (log*-*rank test *P-*value = 0.037, Supplementary Figure 6C).

### Validation of the IGBRS in Different Clinical Subgroups

Although the pathological stage is the most significant factor that influences CRC patient survival, other factors such as sex, age, and location are also important (Loupakis et al., 2015; Thrumurthy et al., 2016; Guo et al., 2019; Siegel et al., 2020); therefore, we stratified CRC patients in the training cohort by four clinical characteristics. The results suggested that in all subgroups including males and females, older (age ≥65 years) and younger (age <65 years) patients, and distal and proximal, the patients in the high-risk groups had shorter RFS times than those in the low-risk groups (Supplementary Figure 7, *P* < 0.05). More convincingly, similar results were obtained in the validation dataset (Supplementary Figure 8). Additionally, in all CRC patients who underwent chemotherapy, the patients in the high-risk groups had shorter RFS and OS times than those in the low-risk groups (Supplementary Figures 7G,H; *P* < 0.0002). These results suggest that the effects of chemotherapy in high-risk patients were significantly worse than those in low-risk patients. Furthermore, among the subtypes of chemotherapy, including 5FU, FOLFOX, and FUFOL, the RFS and OS of chemotherapy in high-risk patients were significantly worse than those in low-risk patients (Supplementary Figure 9).

### IGBRS Can Predict Clinical Outcomes Independent of TP53, KRAS, or BRAF Mutation Status

In view of the fact that TP53, KRAS, and BRAF are commonly mutated genes in CRC (Russo et al., 2005; Sinicrope et al., 2015; Guo et al., 2019), we analyzed the performance of the IGBRS among patients with different TP53, KRAS, and BRAF mutation statuses. First, we analyzed the proportion of high-risk and low-risk patients with different mutation statuses in the training cohort. The results showed that IGBRS can predict RFS and OS independent of the *TP53*, *KRAS*, or *BRAF* mutation status (Supplementary Figures 10, 11, *P* < 0.01).

### IGBRS Can Predict Clinical Outcomes Independent of CpG Island Methylation Phenotype, Mismatch Repair, and Chromosomal Instability Status

A CpG island methylation phenotype (CIMP) occurs when promoter CpG island methylation causes epigenetic silencing, which is an epigenetic phenotype of CRC, called CIMP cancer (Lao and Grady, 2011). Chromosomal instability (CIN) is a special phenotype of CRC, which is found in the majority of CRC and leads to a different pattern of gene alterations that contribute to tumor formation (Grady, 2004). We analyzed the performance of the IGBRS in patients with different molecular CIMP, mismatch repair (MMR), and CIN subtypes. The results suggested that IGBRS can predict clinical outcomes independent of the CIMP, MMR, and CIN status, validating the IGBRS (Supplementary Figures 12A–F, 13A–F, *P* < 0.05).

Moreover, compared with the CIN-positive group, the CIN-negative group had a higher risk score (Supplementary Figure 12H, *P* < 0.01), while compared with the high-risk group, CIN-positive patients were obviously concentrated in the low-risk group (Supplementary Figure 12G, 31% vs. 15%, *P* < 0.01), which may have something to do with the role of CIN in triggering the immune response. Among the MMR-proficient (pMMR) and MMR-deficient (dMMR) subgroups, patients in the dMMR subgroup had a lower risk score than those in the pMMR subgroup (Supplementary Figure 13H, *P* < 0.0001). Furthermore, the higher risk score was obviously concentrated in the CIMP-positive group compared with the CIMP-negative group. Besides, compared with the low-risk group, CIMP-positive patients were obviously concentrated in the high-risk group (Supplementary Figure 12G, 87% vs. 75%, *P* < 0.01).

### Immune Cell Infiltration and Immune Checkpoint Correlates of the IGBRS

**FIGURE 6:**
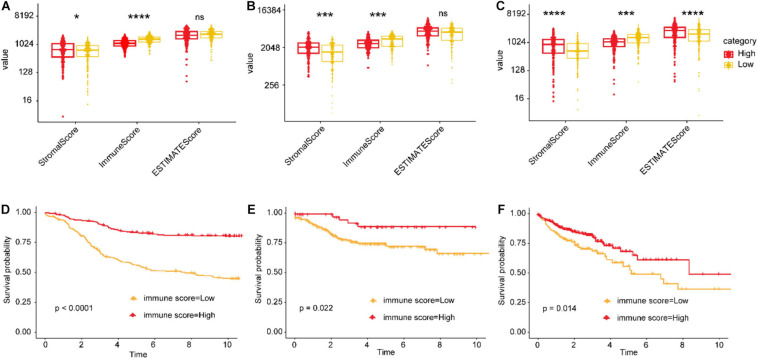
The relationship between immune infiltration scores and risk scores and recurrence. **(A,B)** Differences in the stromal, immune, and ESTIMATE scores (the ESTIMATE score can be obtained by adding the immune score and the stromal score, which can be used to estimate tumor purity) between the high- and low-risk groups in the training, test, and TCGA cohorts. **(C–F)** Impact of the immune score on RFS of CRC patients based on Kaplan–Meier analysis in the training, test, and TCGA cohorts. The ordinate represents the values of the three scores. *FDR < 0.05, **FDR < 0.01, ***FDR < 0.001, and ****FDR < 0.0001. TCGA, The Cancer Genome Atlas; CRC, colorectal adenocarcinoma; RFS, recurrence-free survival; FDR, false discovery rate.

Tumor microenvironments contain a variety of cell types, including immune cells, interstitial cells, endothelial cells, and inflammatory mediators, and extracellular matrix (ECM) molecules (Runa et al., 2017). To explore the potential mechanisms underlying the association between the IGBRS and CRC recurrence, we used a series of analysis methods related to the immune profile. In the training, test, and TCGA cohorts, the immune score of the high-risk group was significantly lower than that of the low-risk group (Figures 6A–C). Kaplan–Meier analysis of the CRC data in the three cohorts showed that RFS was significantly shorter in the low-immune score groups (Figures 6D–F).

Since risk scores are closely related to immune infiltration scores, we analyzed specific immunophenotypic differences between high- and low-risk groups, including immune cell infiltration and immune checkpoints. The CIBERSORT package was used to assess the proportions of 22 immune cell subtypes based on the expression profile. CRC samples with *P* < 0.05 in the CIBERSORT analysis results were used for further analyses. There were large differences in the proportions of infiltrating immune cells between the high- and low-risk score groups in the training and test cohorts, which were predominantly M1 macrophages, M2 macrophages, memory CD4 T cells, regulatory T cells, and CD8 T cells (Supplementary Figures 14A,B). Moreover, we calculated the Pearson correlation coefficients of seven IRGs with various immune cells in the training and test cohorts (Supplementary Figures 14C,D). Significant correlations were found in both matrices, especially for GZMB and CXCL3, which were significantly associated with CD8 T cells (*r* = 0.18, *P* < 0.01; *r* = −0.17, *P* < 0.01). When further restricted to immune cells for the TCGA cohort, we obtained similar results (Supplementary Figure 15).

Next, we extended the analysis to 49 immune checkpoint molecules, and the results for the training and test cohorts are shown in Supplementary Figures 16A,B, respectively. In total, we found that the expression of 13 immune checkpoints [*CD274* (*PD-L1*), *CTLA4*, *ICOS*, *BTLA*, *CD27*, *TNFRSF14*, *TNFRSF18*, *TNFRSF9*, *CD28*, *CD80*, *IDP1*, *LAG3*, and *TIGIT*] was significantly downregulated in patients with high-risk scores in both cohorts (Supplementary Figures 16C,D; FDR < 0.05). In addition, we calculated the Pearson correlation coefficients of seven IRGs with various differentially expressed immune checkpoints (Supplementary Figure 17). Good correlations were found in both matrices, especially for *GZMB*, *LGR5*, and *PLAU*, which were significantly associated with many differentially expressed immune checkpoints. Surprisingly, CD274 was significantly positively correlated with *GZMB*, *PLAU*, *CXCL3*, and *IL1R2*, while it was significantly negatively correlated with *LGR5* in the training and test cohorts. When further restricted to immune checkpoints for the TCGA cohort, we obtained similar results (Supplementary Figure 18).

In terms of guidance for immunotherapy, we studied the application of the signature in cutaneous melanoma (TCGA-SKCM). The results showed that IGBRS can also be used to predict the prognosis of patients with cutaneous melanoma (Supplementary Figures 19A,B). The effects of immunotherapy in cutaneous melanoma patients with high-risk score were weaker than those in patients with low-risk scores (5-year OS: 57% vs. 88%, Supplementary Figure 19C), and ROC analysis showed that our signature was used to predict the 4-year survival rate of immunotherapy patients with an AUC value as high as 0.700 (Supplementary Figure 19D). In addition, when our signature was applied to the cohort of anti-CTLA4 immunosuppressant therapy (GSE63557) and the anti-MAGE-A3 immunosuppressant therapy cohort (GSE35640), we found that the risk score of immunotherapy responders was significantly lower than that of non-responders (Supplementary Figures 19E,F).

### Tumor Somatic Mutation Correlates of the IGBRS

**FIGURE 7:**
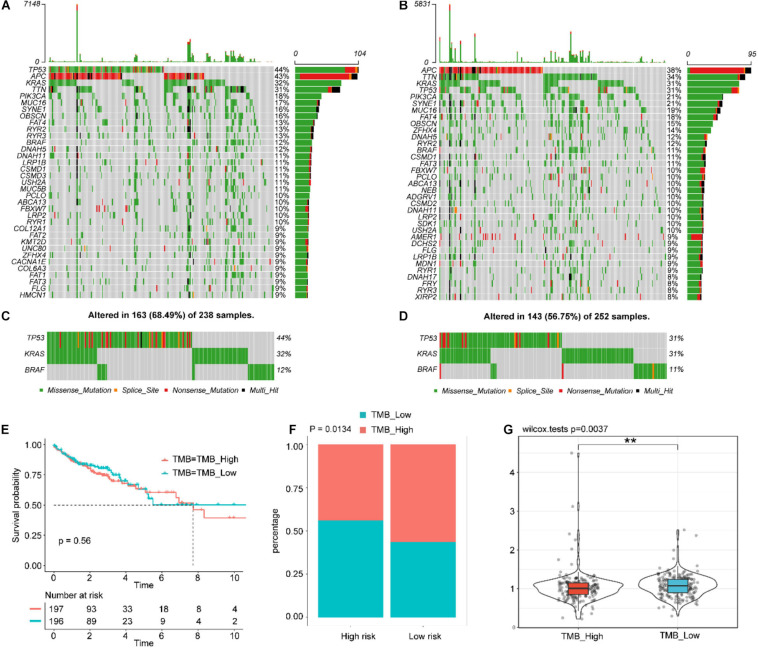
Tumor somatic mutational landscape of the IGBRS. **(A)** The top 35 gene mutations in the high-risk score group. **(B)** The top 35 gene mutations in the low-risk score group. **(C)** and **(D)** Mutations in the commonly mutated genes *TP53*, *BRAF*, and *KRAS* in the high-risk and low-risk score groups, respectively. **(E)** Kaplan-Meier curves of OS in all CRC patients based on TMB. **(F)** Difference in TMB between the high- and low-risk groups. **(G)** Difference in risk scores between the high and low TMB groups. Each gray block indicates that the gene does not have any form of mutation in the sample. Each gray block represents that the gene does not have any form of mutation in the sample. IRG, immune-related gene; IGBRS, IRG-based recurrence signature. **p < 0.01.

We analyzed the differences with respect to the distribution of somatic mutations between low- and high-risk score groups in the TCGA-COAD cohort using the maftools package. Supplementary Figure 20A shows the overall gene mutations in the TCGA-COAD cohort. Somatic mutations appeared in 390 (97.74%) of 399 samples. Figures 7A,B shows the top 30 gene mutations in the high- and low-risk score groups, respectively. The commonly mutated genes *TP53*, *BRAF*, and *KRAS* were altered in 311 (77.94%) of 399 samples (Supplementary Figure 20B), especially TP53 (Figures 7C,D). The Kaplan–Meier curves for OS suggested that there was no statistical difference in survival between the high- and low-TMB groups (log-rank test *P*-value = 0.56, Figure 7E). As shown in Figure 7F, the low-risk score group presented more extensive TMB than the high risk-score group (*P* = 0.0134). By contrast, compared with the high-TMB group, the low-TMB group had a higher risk score (Figure 7G, *P* = 0.0037). Moreover, we explored the mutation landscape of the seven IGRs in the IGBRS (Supplementary Figure 20C). Their mutation rates were very low and were well suited as diagnostic or prognostic biomarkers.

### Identification of IGBRS as an Independent Risk Factor for CRC Patients

With the training cohort, we performed univariate and multivariate Cox regression analyses for each clinical factor (Table 1) and screened factors with *P* < 0.05, which included the TNM stage, KRAS status, and the IGBRS. The multivariate modeling results showed that several patient and disease variables were significantly associated with survival, including a *KRAS* mutation (HR = 1.54, *P* = 0.0245), stage III (HR = 10.17, *P* = 0.0239), stage IV (HR = 12.78, *P* = 0.0178), and increasing risk score (HR = 2.71, *P* < 0.0001). The IGBRS was a significant independent predictor of RFS in CRC patients. Additionally, the results for the test (Supplementary Table 4, HR = 3.84, *P* < 0.0001) and validation (Supplementary Table 5, HR = 1.58, *P* = 0.0009) cohorts confirmed the independent predictive value of the IGBRS for RFS in CRC patients. In addition, as shown in Table 1, the IGBRS was an independent factor for OS (HR = 2.42, *P* < 0.0001). As expected, in the GSE17538 (Supplementary Table 6, HR = 2.18, *P* = 0.0011) and TCGA (Supplementary Table 7, HR = 2.00, *P* = 0.0065) cohorts, the IGBRS was still found to be an independent factor for OS after multivariate Cox regression analysis. In addition, the results for the test cohort showed that the IGBRS was an independent factor for DSS (Supplementary Table 4, HR = 3.09, *P* < 0.0001).

**TABLE 1:** Univariable and multivariable Cox regression analysis of IGBRS and characteristics with RFS and OS in GSE39582.

Variable	Relapse-free survival						Overall survival					
	Univariate cox			Multivariate cox			Univariate cox			Multivariate cox		
	*p*-value	HR	95% CI	*p*-value	HR	95% CI	*p*-value	HR	95% CI	*p*-value	HR	95% CI
**Age**												
>65 Vs. ≤65 (reference)	0.9150	1.0180	0.7296–1.4210	0.4706	1.5273	0.8056–1.6270	0.0138	1.4620	1.0800–1.9780	<0.0001	1.7394	1.2626–2.3960
**Sex**												
Male Vs. female (reference)	0.1410	1.2840	0.9206–1.7900	0.0968	0.9815	0.9482–1.9160	0.0684	1.3100	0.9799–1.7500	0.0758	1.3111	0.9722–1.7680
**TNM stage**												
**I (reference)**												
II	0.0393	7.9980	1.1070–57.7800	0.0814	5.8670	0.7998–42.6740	0.2980	1.5550	0.6772–3.5700	0.4615	1.3804	0.5853–3.2560
III	0.0054	16.3930	2.2810–117.8100	0.0239	10.1700	1.3776–73.1500	0.1280	1.9130	0.8299–4.4120	0.2490	1.6627	0.7005–3.9460
IV	0.0091	15.3930	1.9900–127.5000	0.0178	12.7800	1.5563–103.7330	<0.0001	7.8110	3.3021–18.4750	<0.0001	7.1807	2.9121–17.060
**Location**												
Proximal vs. distal (reference)	0.8416	0.3200	0.5993–1.1820	0.2340	0.7871	0.5333–1.1690	0.6510	1.0700	0.7988–1.4320	0.9041	1.0206	0.7323–1.4230
**MMR status**												
dMMR Vs. pMMR (reference)	0.0112	0.4345	0.2282–0.8272	0.6168	0.8076	0.3498–1.8600	0.3038	0.7739	0.4748–1.2610	0.6928	1.1328	0.6103–2.1020
**CIMP**												
Positive vs. negative (reference)	0.4220	0.8092	0.4827–1.3570	0.7896	0.9077	0.4454–1.8470	0.7860	1.0570	0.7077–1.5790	0.4663	1.2375	0.6975–2.1960
**CIN**												
Positive vs. negative (reference)	0.3020	1.3170	0.7808–2.2220	0.6872	0.8826	1.0516–4.6260	0.1880	0.7767	0.5331–1.1320	0.3441	0.8138	0.5310–1.2470
**TP53 status**												
MUT vs. WT (reference)	0.1570	1.3223	0.8984–1.9460	0.5764	1.1240	0.7455–1.6930	0.3140	1.1960	0.8439–1.6960	0.4809	1.1417	0.7898–1.6510
**KRAS status**												
MUT vs. WT (reference)	0.0774	1.3550	0.9672–1.8980	0.0245	1.5410	1.0567–2.2450	0.0377	1.3597	1.0176–1.8170	0.1583	1.2646	0.9127–1.7520
**BRAF status**												
MUT Vs. WT (reference)	0.9404	1.0240	0.5504–1.9050	0.1250	2.0880	0.8161–5.3380	0.6920	1.1098	0.6628–1.8580	0.5860	0.8085	0.3761–1.7380
**Risk score**												
Increasing	<0.0001	2.8280	1.9780–4.0420	<0.0001	2.7080	1.8515–3.9480	<0.0001	2.5470	1.8820–3.4470	<0.0001	2.4176	1.7628–3.3160

*IBGRS, immune gene-set based recurrence signature; HR, hazard ratio; CI, confidence interval; WT, wild-type; MUT, mutation.*

Moreover, we performed a prognostic meta-analysis to investigate the comprehensive prognostic value across all groups. The results indicated that the IGBRS was a significant risk factor for RFS (*n* = 1,386, combined HR = 2.85, 95% CI = 1.77–4.58, *P* < 0.01) and OS (*n* = 1,375, combined HR = 2.48, 95% CI = 1.72–3.58, *P* < 0.01) in CRC patients (Supplementary Figures 21A,B).

### Gene Set Enrichment Analysis (GSEA)

We also identified pathways that were up- and downregulated between the high- and low-risk score groups by running a GSEA of the adjusted expression data for all transcripts. GSEA identified that, compared with the high-risk score group, the genes highly expressed in the low-risk score group were significantly enriched in 15 pathways including the cell cycle, MMR, nucleotide excision repair, cytokine–receptor interaction, ECM–receptor interaction, cell adhesion molecules (CAMs), and DNA replication (Supplementary Figures 22A,B,D). Besides, compared with the patients with low-risk scores, the genes with significantly higher expression in the high-risk score group were mainly concentrated in pathways involved in cancer, the MAPK signaling pathway, and the P53 signaling pathway (Supplementary Figure 22C).

## Discussion

Several lines of evidence have demonstrated that IRGs have indispensable roles in inflammation and innate immunity and antitumor effects. Considering the close relationships between the immune system and the occurrence and development of CRC (Ferrone and Dranoff, 2010), we believe that it is imperative to develop biomarkers related to RFS and OS for CRC. To this end, we analyzed the relationships between IGBRS and CRC patient recurrence for the first time. Using large-scale datasets from multiple centers, we identified an IGBRS based on seven IRGs that were significantly associated with RFS and OS in CRC patients. In addition, the prognostic value of the IGBRS in different molecular and clinical subtypes was verified, and this feature was related to different immune and somatic mutation landscapes. Ultimately, we concluded that the signature is an independent risk factor in CRC patients.

In the present study, we confirmed that IRGs were strongly correlated with recurrence based on unsupervised cluster analysis of 566 CRC patients from the GSE39582 dataset. Seven IRGs (BMP4, CXCL3, IL1R2, LGR5, GZMB, PTGDR, and PLAU) were applied to construct a recurrence signature for CRC. To avoid false positives in the sequencing data, the 867 CRC patients in the qRT-PCR, test, and validation cohorts were used to validate the stability of the IGBRS. In addition, through analysis of the training, qRT-PCR, validation, and TCGA cohorts, we found that the IRG combination accurately predicted OS and DSS in CRC patients. Finally, we conducted a meta-analysis based on four cohorts with RFS and OS data to fully understand the value of IGBRS in assessing the prognosis of CRC.

We have developed and tested a new prognostic IRG signature, which can carry out risk stratification and predict the OS and recurrence of patients with CRC more accurately than the AJCC stage. The effect of chemotherapy in high-risk patients (3-year OS: 35% in the training cohort and 5-year OS: 43% in the qRT-PCR cohort) was significantly weaker than that in the low-risk group (3-year OS: 70% in the training cohort and 5-year OS: 85% in the qRT-PCR cohort). Among different chemotherapy subtypes, the RFS and OS after single use of capecitabine, 5FU, FOLFOX, FUFOL, or XELOX (combined capecitabine with oxaliplatin) scheme in high-risk patients were significantly worse than those in low-risk patients. In terms of guidance for immunotherapy, the results showed that the effects of immunotherapy in cutaneous melanoma patients with high-risk score were weaker than those in patients with low-risk scores (5-year OS: 57% vs. 88%). In addition, we found that the risk score of immunotherapy responders was significantly lower than that of non-responders in the cohort of anti-CTLA4 immunosuppressant therapy (GSE63557) and the anti-MAGE-A3 immunosuppressant therapy cohort (GSE35640). This has great significance for the guidance of clinical stratified treatment. Patients with low-risk scores can appropriately consider adjuvant chemotherapy and immunotherapy before and after operation. Besides, patients with high-risk scores need to consider total surgical resection and radiotherapy more actively and re-check more frequently to monitor the recurrence for further early treatment.

Pathological staging is currently the most important factor in the clinical evaluation of CRC prognosis. Different stages of CRC have different immune statuses and respond differently to immunotherapy (Brahmer et al., 2012). We explored the applicability of our signature in different staging subgroups. As expected, the signature performed well in both early and advanced stages. Age, sex, and location were also important factors affecting the prognosis of patients with CRC (Loupakis et al., 2015; Thrumurthy et al., 2016; Guo et al., 2019; Siegel et al., 2020). Patients with CRC who are aged younger than 50 years have higher 5-year relative survival rates than their older counterparts at every stage of diagnosis (Siegel et al., 2020). Besides, the signature performed convincingly well in all subgroups including males and females, older (age ≥65 years) and younger (age <65 years) patients, and distal and proximal metastases. These findings indicate that our signature may help to identify high-risk CRC patients independent of other clinical factors affecting prognosis and may better guide clinical treatment.

TP53, KRAS, and BRAF are commonly mutated oncogenes in CRC (Russo et al., 2005; Sinicrope et al., 2015; Guo et al., 2019). We analyzed the performance of IGBRS among patients with different TP53, KRAS, and BRAF mutation statuses. The results suggest that the risk stratification effect of IGBRS is independent of these oncogenes. Then, we analyzed the differences with respect to the distribution of somatic mutations between the low- and high-risk groups in the TCGA-COAD cohort. The results indicated that the low-risk group presented more extensive TMB than the high-risk group.

CIN, CIMP, and microsatellite-unstable (MSI) are the three molecular types of CRC. MMR refers to the repair mechanism to restore the normal nucleotide sequence in DNA molecules containing mismatch bases, which is mainly used to correct the mismatched base pairs in the double helix of DNA. MSI refers to the phenomenon that when the function of MMR is abnormal, the replication errors of microsatellites cannot be corrected and accumulate, changing the length or composition of microsatellite sequences. We analyzed the performance of the IGBRS among patients with different CIN, MMR, and CIMP statuses. The results showed that compared with the CIN-positive group, the CIN-negative group had a higher risk score and a higher proportion of high-risk patients and showed better RFS and OS. Among the pMMR and dMMR subgroups, the patients in the high-risk group had shorter RFS and OS times than those in the low-risk group. Patients in the dMMR subgroup had lower risk scores than those in the pMMR subgroup. In addition, higher risk scores and high-risk patients were more concentrated in the CIMP-positive group than in the CIMP-negative group, which was associated with worse RFS and OS. A number of studies showed that lower 5-year survival rates were observed in microsatellite-stable cancer patients with CIMP-low or CIMP-high status than in patients with no CIMP (Barault et al., 2008; Sinicrope et al., 2015), which strongly confirmed the ability of the IGBRS to conduct risk stratification of CRC patients.

The present study found that this signature was a good predictor of the outcomes of several cohorts and subgroups; therefore, we examined possible underlying mechanisms. The degree of immune infiltration significantly affects the prognosis of CRC (Mao et al., 2018). In this study, the high-risk group had significantly lower immune scores than the low-risk group, and the patients with high immune scores tended to have better prognoses. The increased accumulation of CD8 T cells, regulatory T cells, and proinflammatory macrophages (M1) and the reduced accumulation of immunosuppressive macrophages (M2) indicated better prognosis for CRC (Funada et al., 2003; Correale et al., 2012). High-risk patients were characterized by high proportions of M2 macrophages and low proportions of activated memory CD4 T cells, CD8 T cells, regulatory T cells, and M1 macrophages, suggesting that the IRGs included in our combination may affect prognosis by interacting with infiltrating immune cells. Therefore, we also calculated the Pearson correlation coefficients of seven IRGs with various immune cells. Good correlations were found in both matrices, especially for *GZMB*, which is significantly associated with a variety of immune cells.

We further found that the two groups of patients had different characteristics of immune checkpoints. The *CD274* (*PD-L1*), *CTLA4*, *ICOS*, *BTLA*, *CD27*, *TNFRSF14*, *TNFRSF18*, *TNFRSF9*, *CD28*, *CD80*, *IDP1*, *LAG3*, and *TIGIT* levels were significantly downregulated in patients with high-risk scores. The PD-1 plus CTLA4 blockade is highly effective in advanced-stage, dMMR CRC, yet not in pMMR tumors (Chalabi et al., 2020). Responses to immune checkpoint inhibitors correlated with PD-L1 expression (Salem et al., 2018). Studies have demonstrated that relatively high PD-L1 expression in cancer cells was associated with a good prognosis in CRC patients (Wyss et al., 2019; Noh et al., 2020). The high expression of *PD-L1* and *CTLA4* in the low-risk group not only demonstrated that the IGBRS is an efficient classifier of risk stratification in CRC and closely related to tumor immunity but also suggested that the IGBRS may be a reference for the classification of CRC patients treated with immune checkpoint inhibitors. To further verify this possibility, we analyzed the correlations of seven IRGs and immune checkpoints for differential expression in each cohort. *CD274* was significantly positively correlated with *GZMB*, *PLAU*, *CXCL3*, and *IL1R2*, while it was significantly negatively correlated with *LGR5* in the training and test cohorts.

All of the genes in the IGBRS were found to be involved in CRC progression (Kalmár et al., 2015; Mar et al., 2015; D’Eliseo et al., 2016; Morgan et al., 2018; Zhou et al., 2018; Liao et al., 2019; Lin et al., 2019). Bone morphogenetic protein 4 (*BMP4*) encodes a secreted ligand of the transforming growth factor-beta (TGF-β) superfamily of proteins. Deng et al. (2009) and Zhou et al. (2018) found that *BMP4* knockdown could ameliorate CRC cell migration, and invasion and overexpression of *BMP4* enhance the invasiveness of Smad4-deficient human CRC cells. The findings of Yokoyama et al. (2017) suggest inhibition of autocrine BMP4 as a candidate treatment strategy for CRC. In our study, *BMP4* expression (HR = 1.213, *P* = 0.02) was an independent risk factor for the prognosis of patients with CRC and was positively correlated with the infiltration abundance of M2 macrophages in tumor tissue (*r* = 0.28, *P* < 0.0001). The above findings suggest that BMP4 may be secreted by M2 macrophages and participate in the malignant biological behavior of CRC cells.

The plasminogen activator urokinase (*PLAU*) encodes a secreted serine protease that converts plasminogen to plasmin and acts as one of the TGF-β downstream factors. Lin et al. (2019) reported that inhibition of PLAU can suppress CRC cell proliferation and progression. In the present study, *PLAU* (HR = 1.357, *P* = 0.001) also acted as an independent risk factor for the prognosis of patients with CRC and was negatively correlated with the abundance of infiltrating regulatory T cells in tumor tissue (*r* = −0.26, *P* < 0.0001). Moreover, *PLAU* was positively correlated with the expression of many immune checkpoint genes, including *CD274* and *CALT4*, and may act as a key molecule for tumor cells to escape immune surveillance.

Granzyme B (*GZMB*) (HR = 0.742, *P* < 0.001), whose encoded preproprotein is secreted by natural killer cells and cytotoxic T-lymphocytes, also acted as an independent protective factor for the prognosis of patients with CRC. Koelzer et al. (2017) found that the immune-activated phenotype was associated with high counts of intratumoral CD8 cytotoxic T-lymphocytes (*P* = 0.007) and the expression of the immune effector molecule GZMB (*P* < 0.001). The increased infiltration of cytotoxic T-lymphocytes is the key to tumor immune rejection. Our study suggests a significant positive correlation between the expression of *GZMB* and the abundance of infiltrating cytotoxic T-lymphocytes in each cohort (*r* = 0.21, *P* < 0.001). The significant downregulation of *GZMB* expression in CRC patients with low risk scores suggests that GZMB may kill tumor cells and inhibit their malignant biological behavior to improve the prognosis of CRC patients by activating tumor immune rejection.

The antimicrobial gene C-X-C motif chemokine ligand 3 (*CXCL3*) encodes a member of the CXC subfamily of chemokines. The encoded protein is a secreted growth factor that signals through the G protein-coupled receptor CXC receptor 2 and plays a role in inflammation and as a chemoattractant for neutrophils. Liao et al. (2019) demonstrated that KRAS^∗^-mediated repression of IRF2 results in high expression of CXCL3, which binds to CXCR2 on myeloid-derived suppressor cells and promotes their migration to the tumor microenvironment, which drives immune suppression and immune therapy resistanc in CRC. Our results suggest a positive correlation (*r* = 0.31, *P* < 0.0001) between the mRNA expression levels of *CXCL3* and *CD274*, which encodes PD-L1, and a negative correlation (*r* = −0.17, *P* < 0.0001) between the mRNA expression levels and the abundance of infiltrating cytotoxic T-lymphocytes. These results suggest that CXCL3 may transmit inhibitory signals through the CXCL3–CXCR2 axis, reduce the proliferation of CD8 T cells in lymph nodes, and promote immune escape. CXCL3 may act as one of the targets to enhance immune efficacy.

The protein encoded by *LGR5* is a leucine-rich repeat containing receptor (LGR) and member of the G protein-coupled, seven-transmembrane receptor (GPCR) superfamily. The encoded protein is a receptor for R-spondins and is involved in the canonical Wnt signaling pathway (Glinka et al., 2011). It has been demonstrated that LGR5-positive cancer cells functionally act as stem cells in human CRCs (Fumagalli et al., 2020). Jang et al. (2018) found that LGR5 overexpression attenuates proliferation, migration, and colony formation in CRC cells. Besides, LGR5 functions as a tumor suppressor in the late stages of CRC progression and is an independent prognostic marker for better clinical outcomes in CRCs. In the present study, LRG5 (HR = 0.855, *P* = 0.003) also acted as an independent protective factor for the prognosis of patients with CRC.

However, the contributions of prostaglandin D2 receptor (PTGDR) and interleukin 1 receptor type 2 (IL1R2) to CRC immune microenvironment remodeling remain unknown. In the present study, these genes showed strong correlations with tumor immune cell infiltration and immune checkpoints, but these correlations require further exploration. Although the prospects for IRG signatures are promising, they also have certain limitations. On the one hand, all cohorts were retrospective, and this risk scoring system still needs to be prospectively verified. On the other hand, because of the high spatial heterogeneity of the tumor immune microenvironment, the relationships between the IGBRS and immune cell infiltration and immune checkpoints are based on estimates of tumor characteristics, which may lead to errors. Further research is needed to verify our findings.

## Conclusion

In general, we developed and tested a new recurrence immune-related gene signature for CRC. Our research provides new insights into the link between immunotherapy and CRC. This IGBRS may help clinicians develop personalized treatment plans, especially when choosing which patients will benefit from immunotherapy, and may improve the survival of CRC patients.

## Data Availability Statement

The original contributions generated for this study are included in the article/Supplementary Material, further inquiries can be directed to the corresponding author/s.

## Ethics Statement

The studies involving human participants were reviewed and approved by the Ethics Committee/Institutional Review Board of the Cancer Institute/Hospital, Peking Union Medical College and Chinese Academy of Medical Sciences (approval no. NCC2013RE-025). The patients/participants provided their written informed consent to participate in this study.

## Author Contributions

RM, TZ, and YL conceived the project and designed the experiments. ZW, FY, and RM carried out the experiments. RM and FY contributed equally to this work. YL, TZ, and RM wrote the manuscript. RM, CX, and QL carried out the statistical analysis and gave assistance in collecting tissue samples. TZ contributed to manuscript revision. All authors provided suggestions during manuscript preparation and read the final version.

## Conflict of Interest

The authors declare that the research was conducted in the absence of any commercial or financial relationships that could be construed as a potential conflict of interest.

## Footnotes

1 https://www.ncbi.nlm.nih.gov/gds/

2 https://portal.gdc.cancer.gov/

3 https://www.immport.org/home

4 www.broadinstitute.org/gsea

## Abbreviations

CRC: colorectal adenocarcinoma
TCGA: The Cancer Genome Atlas
GEO: Gene Expression Omnibus
ROC: receiver operating characteristic
OS: overall survival
AUC: area under the curve.

## References

[B1] Barault L. Charon-Barra C. Jooste V. de la Vega M. F. Martin L. Roignot P. (2008). Hypermethylator phenotype in sporadic colon cancer: study on a population-based series of 582 cases. *Cancer Res.* 68 8541–8546. 10.1158/0008-5472.can-08-1171 18922929

[B2] Brahmer J. R. Tykodi S. S. Chow L. Q. Hwu W. J. Topalian S. L. Hwu P. (2012). Safety and activity of anti-PD-L1 antibody in patients with advanced cancer. *N. Engl. J. Med.* 366 2455–2465. 22658128 10.1056/NEJMoa1200694 PMC3563263

[B3] Chalabi M. Fanchi L. F. Dijkstra K. K. Van den Berg J. G. Aalbers A. G. Sikorska K. (2020). Neoadjuvant immunotherapy leads to pathological responses in MMR-proficient and MMR-deficient early-stage colon cancers. *Nat. Med.* 26 566–576. 10.1038/s41591-020-0805-8 32251400

[B4] Chrétien S. Zerdes I. Bergh J. Matikas A. Foukakis T. (2019). Beyond PD-1/PD-L1 inhibition: what the future holds for breast cancer immunotherapy. *Cancers* 11:628. 10.3390/cancers11050628 31060337 PMC6562626

[B5] Correale P. Rotundo M. S. Botta C. Del Vecchio M. T. Ginanneschi C. Licchetta A. (2012). Tumor infiltration by T lymphocytes expressing chemokine receptor 7 (CCR7) is predictive of favorable outcome in patients with advanced colorectal carcinoma. *Clin. Cancer Res.* 18 850–857. 10.1158/1078-0432.ccr-10-3186 22142823

[B6] Curran M. A. Montalvo W. Yagita H. Allison J. P. (2010). PD-1 and CTLA-4 combination blockade expands infiltrating T cells and reduces regulatory T and myeloid cells within B16 melanoma tumors. *Proc. Natl. Acad. Sci. U.S.A.* 107 4275–4280. 10.1073/pnas.0915174107 20160101 PMC2840093

[B7] D’Eliseo D. Di Rocco G. Loria R. Soddu S. Santoni A. Velotti F. (2016). Epitelial-to-mesenchimal transition and invasion are upmodulated by tumor-expressed granzyme B and inhibited by docosahexaenoic acid in human colorectal cancer cells. *J. Exp. Clin. Cancer Res.* 35:24. 10.1186/s13046-016-0302-6 PMC4736710 26830472

[B8] Deng H. Ravikumar T. S. Yang W. L. (2009). Overexpression of bone morphogenetic protein 4 enhances the invasiveness of Smad4-deficient human colorectal cancer cells. *Cancer Lett.* 281 220–231. 10.1016/j.canlet.2009.02.046 19321257

[B9] Dupaul-Chicoine J. Arabzadeh A. Dagenais M. Douglas T. Champagne C. Morizot A. (2015). The Nlrp3 inflammasome suppresses colorectal cancer metastatic growth in the liver by promoting natural killer cell tumoricidal activity. *Immunity* 43 751–763. 10.1016/j.immuni.2015.08.013 26384545

[B10] Ferrone C. Dranoff G. (2010). Dual roles for immunity in gastrointestinal cancers. *J. Clin. Oncol.* 28 4045–4051. 10.1200/jco.2010.27.9992 20644090 PMC4872327

[B11] Fumagalli A. Oost K. C. Kester L. Morgner J. Bornes L. Bruens L. (2020). Plasticity of Lgr5-negative cancer cells drives metastasis in colorectal cancer. *Cell Stem Cell* 26 569–578.e7. 10.1016/j.stem.2020.02.008 32169167 PMC7118369

[B12] Funada Y. Noguchi T. Kikuchi R. Takeno S. Uchida Y. Gabbert H. E. (2003). Prognostic significance of CD8+ T cell and macrophage peritumoral infiltration in colorectal cancer. *Oncol. Rep.* 10 309–313. 12579264

[B13] Glinka A. Dolde C. Kirsch N. Huang Y. L. Kazanskaya O. Ingelfinger D. (2011). LGR4 and LGR5 are R-spondin receptors mediating Wnt/β-catenin and Wnt/PCP signalling. *EMBO Rep.* 12 1055–1061. 10.1038/embor.2011.175 21909076 PMC3185347

[B14] Grady W. M. (2004). Genomic instability and colon cancer. *Cancer Metast. Rev.* 23 11–27. 10.1023/a:1025861527711 15000146

[B15] Guo T. A. Wu Y. C. Tan C. Jin Y. T. Sheng W. Q. Cai S. J. (2019). Clinicopathologic features and prognostic value of KRAS, NRAS and BRAF mutations and DNA mismatch repair status: a single-center retrospective study of 1,834 Chinese patients with Stage I-IV colorectal cancer. *Int. J. Cancer* 145 1625–1634. 10.1002/ijc.32489 31162857 PMC6771586

[B16] Hochberg Y. Benjamini Y. (1990). More powerful procedures for multiple significance testing. *Stat. Med.* 9 811–818. 10.1002/sim.4780090710 2218183

[B17] Janakiram M. Chinai J. M. Fineberg S. Fiser A. Montagna C. Medavarapu R. (2015a). Expression, clinical significance, and receptor identification of the newest B7 family member HHLA2 protein. *Clin. Cancer Res.* 21 2359–2366. 10.1158/1078-0432.ccr-14-1495 25549724 PMC4433806

[B18] Janakiram M. Chinai J. M. Zhao A. Sparano J. A. Zang X. (2015b). HHLA2 and TMIGD2: new immunotherapeutic targets of the B7 and CD28 families. *Oncoimmunology* 4:e1026534. 10.1080/2162402x.2015.1026534 26405587 PMC4570140

[B19] Jang B. G. Kim H. S. Chang W. Y. Bae J. M. Kim W. H. Kang G. H. (2018). Expression profile of LGR5 and its prognostic significance in colorectal cancer progression. *Am. J. Pathol.* 188 2236–2250. 10.1016/j.ajpath.2018.06.012 30036518

[B20] Juneja V. R. McGuire K. A. Manguso R. T. LaFleur M. W. Collins N. Haining W. N. (2017). PD-L1 on tumor cells is sufficient for immune evasion in immunogenic tumors and inhibits CD8 T cell cytotoxicity. *J. Exp. Med.* 214 895–904. 10.1084/jem.20160801 28302645 PMC5379970

[B21] Kalmár A. Péterfia B. Hollósi P. Galamb O. Spisák S. Wichmann B. (2015). DNA hypermethylation and decreased mRNA expression of MAL, PRIMA1, PTGDR and SFRP1 in colorectal adenoma and cancer. *BMC Cancer* 15:736. 10.1186/s12885-015-1687-x 26482433 PMC4612409

[B22] Koch M. Beckhove P. Op den Winkel J. Autenrieth D. Wagner P. Nummer D. (2006). Tumor infiltrating T lymphocytes in colorectal cancer: tumor-selective activation and cytotoxic activity in situ. *Ann. Surg.* 244 986–992. 10.1097/01.sla.0000247058.43243.7b 17122624 PMC1856622

[B23] Koelzer V. H. Sokol L. Zahnd S. Christe L. Dawson H. Berger M. D. (2017). Digital analysis and epigenetic regulation of the signature of rejection in colorectal cancer. *Oncoimmunology* 6:e1288330. 10.1080/2162402X.2017.1288330 28507795 PMC5414871

[B24] Koyama S. Akbay E. A. Li Y. Y. Herter-Sprie G. S. Buczkowski K. A. Richards W. G. (2016). Adaptive resistance to therapeutic PD-1 blockade is associated with upregulation of alternative immune checkpoints. *Nat. Commun.* 7:10501. 10.1038/ncomms10501 PMC4757784 26883990

[B25] Lao V. V. Grady W. M. (2011). Epigenetics and colorectal cancer. *Nat. Rev. Gastroenterol. Hepatol.* 8 686–700. 22009203 10.1038/nrgastro.2011.173 PMC3391545

[B26] Lefèvre J. H. Mineur L. Cachanado M. Denost Q. Rouanet P. de Chaisemartin C. (2019). The French research group of rectal cancer surgery (GRECCAR), Does A longer waiting period after neoadjuvant radio-chemotherapy improve the Oncological prognosis of rectal cancer?: three years’ follow-up results of the greccar-6 randomized Multicenter trial. *Ann. Surg.* 270 747–754. 10.1097/sla.0000000000003530 31634178

[B27] Liao W. Overman M. J. Boutin A. T. Shang X. Zhao D. Dey P. (2019). KRAS-IRF2 axis drives immune suppression and immune therapy resistance in colorectal cancer. *Cancer Cell* 35 559–572.e7. 10.1016/j.ccell.2019.02.008 30905761 PMC6467776

[B28] Lin M. Zhang Z. Gao M. Yu H. Sheng H. Huang J. (2019). MicroRNA-193a-3p suppresses the colorectal cancer cell proliferation and progression through downregulating the PLAU expression. *Cancer Manag. Res.* 11 5353–5363. 10.2147/CMAR.S208233 31354344 PMC6578599

[B29] Loupakis F. Yang D. Yau L. Feng S. Cremolini C. Zhang W. (2015). Primary tumor location as a prognostic factor in metastatic colorectal cancer. *J. Natl. Cancer Inst.* 107:dju427. 10.1093/jnci/dju427 PMC4565528 25713148

[B30] Mao Y. Feng Q. Zheng P. Yang L. Zhu D. Chang W. (2018). Low tumor infiltrating mast cell density confers prognostic benefit and reflects immunoactivation in colorectal cancer. *Int. J. Cancer* 143 2271–2280. 10.1002/ijc.31613 29873076

[B31] Mar A. C. Chu C. H. Lee H. J. Chien C. W. Cheng J. J. Yang S. H. (2015). Interleukin-1 receptor Type 2 Acts with c-Fos to enhance the expression of interleukin-6 and vascular endothelial growth factor a in colon cancer cells and induce angiogenesis. *J. Biol. Chem.* 290 22212–22224. 10.1074/jbc.m115.644823 26209639 PMC4571972

[B32] McLachlan G. J. Bean R. W. Ng S. K. (2017). Clustering. *Methods Mol. Biol.* 1526 345–362. 27896751 10.1007/978-1-4939-6613-4_19

[B33] Miller K. D. Nogueira L. Mariotto A. B. Rowland J. H. Yabroff K. R. Alfano C. M. (2019). Cancer treatment and survivorship statistics, 2019. *CA Cancer J. Clin.* 69 363–385. 31184787 10.3322/caac.21565

[B34] Morgan R. G. Mortensson E. Williams A. C. (2018). Targeting LGR5 in colorectal cancer: therapeutic gold or too plastic. *Br. J. Cancer* 118 1410–1418. 10.1038/s41416-018-0118-6 29844449 PMC5988707

[B35] Mu M. Tang Y. Yang Z. Qiu Y. Li X. Mo W. (2020). Effect of different expression of immune-related lncRNA on colon adenocarcinoma and its relation to prognosis. *Biomed. Res. Int.* 2020:6942740. 10.1155/2020/6942740 PMC7294360 32596355

[B36] Newman A. M. Liu C. L. Green M. R. Gentles A. J. Feng W. Xu Y. (2015). Robust enumeration of cell subsets from tissue expression profiles. *Nat. Methods* 12 453–457. 10.1038/nmeth.3337 25822800 PMC4739640

[B37] Noh B. J. Kwak J. Y. Eom D. W. (2020). Immune classification for the PD-L1 expression and tumour-infiltrating lymphocytes in colorectal adenocarcinoma. *BMC Cancer* 20:58. 10.1186/s12885-020-6553-9 31992245 PMC6986059

[B38] Pentcheva-Hoang T. Simpson T. R. Montalvo-Ortiz W. Allison J. P. (2014). Cytotoxic T lymphocyte antigen-4 blockade enhances antitumor immunity by stimulating melanoma-specific T-cell motility. *Cancer Immunol. Res.* 2 970–980. 10.1158/2326-6066.cir-14-0104 25038199

[B39] Robinson M. D. McCarthy D. J. Smyth G. K. (2010). EdgeR: a bioconductor package for differential expression analysis of digital gene expression data. *Bioinformatics* 26 139–140. 10.1093/bioinformatics/btp616 19910308 PMC2796818

[B40] Romero D. (2016). Immunotherapy: PD-1 says goodbye, TIM-3 says hello. *Nat. Rev. Clin. Oncol.* 13 202–203. 10.1038/nrclinonc.2016.40 26977783

[B41] Runa F. Hamalian S. Meade K. Shisgal P. Gray P. C. Kelber J. A. (2017). Tumor microenvironment heterogeneity: challenges and opportunities. *Curr. Mol. Biol. Rep.* 3 218–229. 10.1007/s40610-017-0073-7 29430386 PMC5802345

[B42] Russo A. Bazan V. Iacopetta B. Kerr D. Soussi T. Gebbia N. (2005). TP53-CRC Collaborative Study Group, The TP53 colorectal cancer international collaborative study on the prognostic and predictive significance of p53 mutation: influence of tumor site, type of mutation, and adjuvant treatment. *J. Clin. Oncol.* 23 7518–7528. 10.1200/jco.2005.00.471 16172461

[B43] Salem M. E. Puccini A. Grothey A. Raghavan D. Goldberg R. M. Xiu J. (2018). Landscape of tumor mutation load, mismatch repair deficiency, and PD-L1 expression in a large patient cohort of gastrointestinal cancers. *Mol. Cancer Res.* 16 805–812. 10.1158/1541-7786.mcr-17-0735 29523759 PMC6833953

[B44] Shen J. Ju Z. Zhao W. Wang L. Peng Y. Ge Z. (2018). ARID1A deficiency promotes mutability and potentiates therapeutic antitumor immunity unleashed by immune checkpoint blockade. *Nat. Med.* 24 556–562. 10.1038/s41591-018-0012-z 29736026 PMC6076433

[B45] Siegel R. L. Miller K. D. Goding Sauer A. Fedewa S. A. Butterly L. F. Anderson J. C. (2020). Colorectal cancer statistics. *CA Cancer J. Clin.* 70 145–164. 32133645 10.3322/caac.21601

[B46] Sinicrope F. A. Shi Q. Smyrk T. C. Thibodeau S. N. Dienstmann R. Guinney J. (2015). Molecular markers identify subtypes of stage III colon cancer associated with patient outcomes. *Gastroenterology* 148 88–99. 10.1053/j.gastro.2014.09.041 25305506 PMC4274188

[B47] Thrumurthy S. G. Thrumurthy S. S. Gilbert C. E. Ross P. Haji A. (2016). Colorectal adenocarcinoma: risks, prevention and diagnosis. *BMJ* 354:i3590. 10.1136/bmj.i3590 27418368

[B48] Tian X. Zhu X. Yan T. Yu C. Shen C. Hu Y. (2017). Recurrence-associated gene signature optimizes recurrence-free survival prediction of colorectal cancer. *Mol. Oncol.* 11 1544–1560. 10.1002/1878-0261.12117 28796930 PMC5664005

[B49] Wang J. Sanmamed M. F. Datar I. Su T. T. Ji L. Sun J. (2019a). Fibrinogen-like protein 1 is a major immune inhibitory Ligand of LAG-3. *Cell* 176 334–347.e12. 30580966 10.1016/j.cell.2018.11.010 PMC6365968

[B50] Wang J. Sun J. Liu L. N. Flies D. B. Nie X. Toki M. (2019b). Siglec-15 as an immune suppressor and potential target for normalization cancer immunotherapy. *Nat. Med.* 25 656–666. 10.1038/s41591-019-0374-x 30833750 PMC7175920

[B51] Ward-Kavanagh L. K. Lin W. W. Šedý J. R. Ware C. F. (2016). The TNF receptor superfamily in co-stimulating and co-inhibitory responses. *Immunity* 44 1005–1019. 10.1016/j.immuni.2016.04.019 27192566 PMC4882112

[B52] Wilkerson M. D. Hayes D. N. (2010). ConsensusClusterPlus: a class discovery tool with confidence assessments and item tracking. *Bioinformatics* 26 1572–1573. 10.1093/bioinformatics/btq170 20427518 PMC2881355

[B53] Wyss J. Dislich B. Koelzer V. H. Galván J. A. Dawson H. Hädrich M. (2019). Stromal PD-1/PD-L1 expression predicts outcome in colon cancer patients. *Clin. Colorectal. Cancer* 18 e20–e38. 30389315 10.1016/j.clcc.2018.09.007

[B54] Xiao Y. Freeman G. J. (2015). A New B7:CD28 family checkpoint target for cancer immunotherapy: HHLA2. *Clin. Cancer Res.* 21 2201–2203. 10.1158/1078-0432.ccr-14-2658 25869386 PMC4433776

[B55] Yang B. Shen J. Xu L. Chen Y. Che Y. Qu X. (2019). Genome-wide identification of a novel eight-lncRNA signature to improve prognostic prediction in head and neck squamous cell carcinoma. *Front. Oncol.* 9:898. 10.3389/fonc.2019.00898 31620361 PMC6759597

[B56] Yokoyama Y. Watanabe T. Tamura Y. Hashizume Y. Miyazono K. Ehata S. (2017). Autocrine BMP-4 signaling is a therapeutic target in colorectal cancer. *Cancer Res.* 77 4026–4038. 10.1158/0008-5472.CAN-17-0112 28611046

[B57] Zhang C. Zhang Z. Li F. Shen Z. Qiao Y. Li L. (2018). Large-scale analysis reveals the specific clinical and immune features of B7-H3 in glioma. *Oncoimmunology* 7:e1461304. 10.1080/2162402x.2018.1461304 30377558 PMC6205005

[B58] Zhou J. Liu H. Zhang L. Liu X. Zhang C. Wang Y. (2018). DJ-1 promotes colorectal cancer progression through activating PLAGL2/Wnt/BMP4 axis. *Cell Death Dis.* 9:865. 10.1038/s41419-018-0883-4 30158634 PMC6115399

