# Supplementary material for: Clinical Significance of a Novel Tumor Progression-Associated Immune Signature in Colorectal Adenocarcinoma
Source: Front Cell Dev Biol. 2021 Feb 25;9:625212. doi: 10.3389/fcell.2021.625212 (PMC7959763; doi:10.3389/fcell.2021.625212)
Supplement: Supplementary file 1 [file Data_Sheet_1.docx]

**Supplementary tables**

| **Table-S1: Basic information of datasets included in this study.** | | | | |
| --- | --- | --- | --- | --- |
| **Accession number /Source** | **Platform** | **Number of  patients** | **Stage** | **Survival data** |
| GEO: GSE14333 | GPL570 | 290 | Duke A/B:100 Duke C/D:152 | RFS |
| GEO: GSE17538 | GPL570 | 244 | I/II:101 III/IV:132 | OS/RFS/DSS |
| GEO: GSE33113 | GPL570 | 91 | I/II:91 III/IV:0 | RFS |
| GEO: GSE39582 | GPL570 | 566 | I/II:297 III/IV:265 | OS/RFS |
| GEO: GSE37892 | GPL570 | 130 | I/II:73 III/IV:57 | RFS |
| GEO: GSE38832 | GPL570 | 122 | I/II:53 III/IV:69 | RFS/DSS |
| TCGA: COAD | Illumina RNAseq | 459 | I/II:254 III/IV:194 | OS |
| National Cancer Center, Chinese Academy of Medical Sciences and Peking Union Medical College | qRT-PCR | 129 | I/II:61 III/IV:68 | OS/RFS |
|  |  |  |  |  |

| **Table S2.**Primer Sequences for qRT-PCR. | | | |
| --- | --- | --- | --- |
| Gene Name | Forward Primer | Reverse Primer | bp |
| BMP4 | 5'-CGGAAGCTAGGTGAGTTCGG-3' | 5'-AGAATCCCATCAGGGACGGA-3' | 105 |
| CXCL3 | 5'-CTGCCCTTACCAGAGCTGAAA-3' | 5'-TCCTTTCCAGCTGTCCCTAGA-3' | 64 |
| GZMB | 5'-GATGCAACCAATCCTGCTTCT-3' | 5'-CGATGATCTCCCCTGAAGGAAAA-3' | 81 |
| IL1R2 | 5'-TGACACCCACATAGAGAGCG-3' | 5'-TCTCAACAGAAGACCCTGGC-3' | 85 |
| LGR5 | 5'-TATGTGCCCCCAAGCTGTTT -3' | 5'-TTTGTTCAGGGCCAAGGTCA -3' | 138 |
| PLAU | 5'-GTCCTCCGGATTCCATCCAC -3' | 5'-TTCATCTACCAGATGCCCGC -3' | 155 |
| PTGDR | 5'-TTCTACCGACGGCACATCAC-3' | 5'-GAACTTCCCGAAGCCCATGA -3' | 108 |
| GAPDH | 5'-GAAAGCCTGCCGGTGACTAA -3' | 3'-GCCCAATACGACCAAATCAGAG -5' | 150 |
| qRT-PCR, quantitative real-time polymerase chain reaction. | | | |

| **Table S3.** The 7 genes in prognostic model in training cohort | | | | | |
| --- | --- | --- | --- | --- | --- |
|  | β | HR | lower .95 | upper .95 | *P-value* |
| BMP4 | 0.1927 | 1.2126 | 1.0303 | 14271 | 0.0204 |
| CXCL3 | -0.1689 | 0.8446 | 0.7498 | 0.9514 | 0.0054 |
| GZMB | -0.2983 | 0.7421 | 0.6524 | 0.8442 | <0.0001 |
| LGR5 | -0.1572 | 0.8545 | 0.7698 | 0.9486 | 0.0032 |
| IL1R2 | -0.2584 | 0.7723 | 0.6659 | 0.8957 | 0.0006 |
| PTGDR | -0.5107 | 0.6001 | 0.3760 | 0.9578 | 0.0323 |
| PLAU | 0.3049 | 1.3565 | 1.1302 | 1.6281 | 0.0011 |

| **Table S4**. Univariable and multivariable Cox regression analysis of IGBRS and characteristics with RFS and DSS in test cohort. | | | | | | | | | | | | | | | |
| --- | --- | --- | --- | --- | --- | --- | --- | --- | --- | --- | --- | --- | --- | --- | --- |
| Variable | Relapse-free survival | | | | | | |  | Disease specific survival | | | | | | |
|  | Univariate cox | | |  | Multivariate cox | | |  | Univariate cox | | |  | Multivariate cox | | |
|  | *p* Value | HR | 95% CI |  | *p* Value | HR | 95% CI |  | *p* Value | HR | 95% CI |  | *p* Value | HR | 95% CI |
| Age |  |  |  |  |  |  |  |  |  |  |  |  |  |  |  |
| >65 VS ≤ 65 | 0.0445 | 0.5724 | 0.3322-0.9865 |  | 0.5434 | 08339 | 0. 4643-1.4980 |  | 0.4230 | 0.8046 | 0.4728-13690 |  | 0.4066 | 1.2893 | 0.7075-2.3500 |
| Sex |  |  |  |  |  |  |  |  |  |  |  |  |  |  |  |
| Male VS Female | 0.2835 | 0.7479 | 0.4398-1.2718 |  | 0.4590 | 0.8066 | 0.4566-1.4250 |  | 0.5330 | 1.1872 | 0.6922-2.0360 |  | 0.4358 | 0.7892 | 0.4351-1.4310 |
| TNM stage |  |  |  |  |  |  |  |  |  |  |  |  |  |  |  |
| I |  |  |  |  |  |  |  |  |  |  |  |  |  |  |  |
| II | 0.0723 | 6.4580 | 0.8446-49.3800 |  | 0.1093 | 5.3501 | 0.6871-41.6590 |  | 0.1375 | 4.8090 | 0.6052-38.2200 |  | 0.3192 | 3.0460 | 0.3403-27.2650 |
| III | 0.0101 | 13.6690 | 1.8637-100.2600 |  | 0.0193 | 10.8994 | 1.4744-80.5720 |  | 0.0206 | 11.1270 | 1.4471-85.5600 |  | 0.0680 | 7.1568 | 0.8644-59.2530 |
| IV | <0.0001 | 77.8380 | 10.4268-581.0800 |  | <0.0001 | 64.2892 | 8.2825-499.0130 |  | <0.0001 | 87.6910 | 11.7015-657.1500 |  | 0.0001 | 63.1021 | 7.7789-511.8820 |
| Grade |  |  |  |  |  |  |  |  |  |  |  |  |  |  |  |
| 1 |  |  |  |  |  |  |  |  |  |  |  |  |  |  |  |
| 2 | 0.4640 | 1.5527 | 0.4782-5.0420 |  | 0.9482 | 0.9601 | 0.2807-3.2840 |  | 0.3515 | 1.7490 | 0.5395-5.6720 |  | 0.9385 | 0.9511 | 0.2658-3.4030 |
| 3 | 0.1130 | 2.8860 | 0.7775-10.7120 |  | 0.3199 | 2.0025 | 0.5097-7.8670 |  | 0.0327 | 3.9380 | 1.1193-13.8580 |  | 0.3397 | 1.9084 | 0.5064-7.1920 |
| Ethnicity |  |  |  |  |  |  |  |  |  |  |  |  |  |  |  |
| black |  |  |  |  |  |  |  |  |  |  |  |  |  |  |  |
| Caucasian | 0.9100 | 1.0694 | 0.3328-3.4370 |  | 0.0641 | 3.2347 | 0.9335-11.2090 |  | 0.2314 | 0.5355 | 0.1925-1.4890 |  | 0.6760 | 1.2808 | 0.4013-4.0890 |
| hispanic | 0.5730 | 1.9224 | 0.1977-18.6900 |  | 0.0978 | 7.8762 | 0.6844-90.6390 |  | 0.6094 | 0.5258 | 0.0447-6.1890 |  | 0.9815 | 0.9537 | 0.0173-52.5980 |
| other | 0.9790 | 0.9802 | 0.2193-4.3820 |  | 0.1982 | 2.9217 | 0.5706-14.9600 |  | 0.0957 | 0.2794 | 0.0624-1.2520 |  | 0.6758 | 0.6977 | 0.1291-3.7710 |
| dataset |  |  |  |  |  |  |  |  |  |  |  |  |  |  |  |
| GSE38832 vsGSE17538 | 0.0030 | 0.3432 | 0.1694-0.6952 |  | / | / | / |  | 0.5260 | 0.8625 | 0.5461-1.3620 |  | / | / | / |
| Risk score |  |  |  |  |  |  |  |  |  |  |  |  |  |  |  |
| Increasing | <0.0001 | 3.8420 | 2.1790-6.7730 |  | <0.0001 | 3.8411 | 2.1372-6.9040 |  | <0.0001 | 3.1710 | 1.9710-5.1020 |  | <0.0001 | 3.0935 | 1.8412-5.1980 |
| IGBRS, immune gene set-based recurrence signature; HR, hazard ratio; CI, confidence interval | | | | | | | | | | | | | | | |

| **Table S5.** Univariable and multivariable Cox regression analysis of IGBRS and characteristics with RFS in validation cohort. | | | | | | | |
| --- | --- | --- | --- | --- | --- | --- | --- |
| Variable | Recurrence-free survival | | | | | | |
|  | Univariate cox | | |  | Multivariate cox | | |
|  | *p* Value | HR | 95% CI |  | *p* Value | HR | 95% CI |
| Age |  |  |  |  |  |  |  |
| >65 VS≤65 | 0.9940 | 1.001 | 0.7706-1.300 |  | 0.7244 | 1.0495 | 0.8024-1.3730 |
| Sex |  |  |  |  |  |  |  |
| Male VS Female | 0.7400 | 1.0450 | 0.8060-1.3550 |  | 0.8121 | 1.0325 | 0.7932-1.3440 |
| TNM stage |  |  |  |  |  |  |  |
| II |  |  |  |  |  |  |  |
| III | <0.0001 | 4.3400 | 2.5540-7.3740 |  | <0.0001 | 6.6668 | 3.0410-14.6150 |
| location |  |  |  |  |  |  |  |
| left |  |  |  |  |  |  |  |
| right | 0.0488 | 1.3336 | 1.0015-1.7758 |  | 0.0924 | 1.2883 | 0.9591-1.7300 |
| rectum | 0.3480 | 1.2464 | 0.7869-1.9743 |  | 0.5092 | 0.8514 | 0.5280-1.3730 |
| duke stage |  |  |  |  |  |  |  |
| A |  |  |  |  |  |  |  |
| B | 0.0689 | 1.4466 | 0.9718-2.1534 |  | 0.1542 | 1.3425 | 0.8024-1.3730 |
| C | 0.6283 | 1.1097 | 0.7281-1.6915 |  | 0.9617 | 0.9895 | 0.8953-2.1030 |
| dataset |  |  |  |  |  |  |  |
| GSE14333 |  |  |  |  |  |  |  |
| GSE33113 | < 0.0001 | 0.2936 | 0.1806-0.4774 |  | / | / | / |
| GSE37892 | < 0.0001 | 0.4106 | 0.2873-0.5866 |  | / | / | / |
| Risk score |  |  |  |  |  |  |  |
| Increasing | 0.0002 | 1.6520 | 1.2710-2.1480 |  | 0.0009 | 1.5791 | 1.2040-2.0710 |
| IGBRS, immune gene set-based recurrence signature; HR, hazard ratio; CI, confidence interval | | | | | | | |

|  | **Table S6.** Univariable and multivariable Cox regression analysis of IGBRS andcharacteristics with OS in GSE17538 cohort. | | | | | | | |
| --- | --- | --- | --- | --- | --- | --- | --- | --- |
| Variable | | Overall survival | | | | | | |
|  |  | Univariate cox | | |  | Multivariate cox | | |
|  |  | *p* Value | HR | 95% CI |  | *p* Value | HR | 95% CI |
| Age | |  |  |  |  |  |  |  |
| >65 VS≤65 | | 0.628 | 1.106 | 0.7360-1.6610 |  | 0.2806 | 1.2707 | 0.8223-1.9640 |
| Sex | |  |  |  |  |  |  |  |
| Male VS Female | | 0.9750 | 1.0060 | 0.6685-1.5150 |  | 0.7506 | 0.9319 | 0.6032-1.4400 |
| TNM stage | |  |  |  |  |  |  |  |
| I | |  |  |  |  |  |  |  |
| II | | 0.2565 | 1.8910 | 0.6293-5.6800 |  | 0.6657 | 1.2923 | 0.4038-4.1360 |
| III | | 0.0325 | 3.1860 | 1.1013-9.2200 |  | 0.1962 | 2.0957 | 0.68246.4360 |
| IV | | <0.0001 | 13.5500 | 4.7788-38.4200 |  | <0.0001 | 8.8912 | 2.9296-26.9840 |
| Grade | |  |  |  |  |  |  |  |
| 1 | |  |  |  |  |  |  |  |
| 2 | | 0.2100 | 1.9090 | 0.6946-5.2470 |  | 0.8865 | 1.0793 | 0.3782-3.0800 |
| 3 | | 0.0100 | 4.1690 | 1.4072-12.3510 |  | 0.2016 | 2.0828 | 0.6754-6.4230 |
| Ethnicity | |  |  |  |  |  |  |  |
| black | |  |  |  |  |  |  |  |
| Caucasian | | 0.2771 | 0.6512 | 0.3005-1.4110 |  | 0.9569 | 0.9776 | 0.4300-2.2220 |
| hispanic | | 0.3702 | 0.3612 | 0.0389-.3500 |  | 0.8103 | 0.7185 | 0.0483-10.6850 |
| other | | 0.0839 | 0.3377 | 0.0986-1.1560 |  | 0.5248 | 0.6556 | 0.1784-2.4090 |
| Risk score | |  |  |  |  |  |  |  |
| Increasing | | <0.0001 | 2.8380 | 1.8290-4.4050 |  | 0.0011 | 2.1750 | 1.3651-3.4650 |
| IGBRS, immune gene-set based recurrence signature; HR, hazard ratio. CI, confidence interval | | | | | | | | |

|  | **Table S7.** Univariable and multivariable Cox regression analysis of IGBRS and characteristics with OS in TCGA cohort. | | | | | | | |
| --- | --- | --- | --- | --- | --- | --- | --- | --- |
| Variable | | Overall survival | | | | | | |
|  |  | Univariate cox | | |  | Multivariate cox | | |
|  |  | *p* Value | HR | 95% CI |  | *p* Value | HR | 95% CI |
| Age | |  |  |  |  |  |  |  |
| >65 VS≤65 | | 0.0214 | 1.6560 | 1.0780-2.5450 |  | 0.0030 | 2.1060 | 1.2879-3.4440 |
| Sex | |  |  |  |  |  |  |  |
| Male VS Female | | 0.5260 | 1.1380 | 0.7628-1.6980 |  | 0.5132 | 1.1561 | 0.7486-1.7850 |
| TNM stage | |  |  |  |  |  |  |  |
| I | |  |  |  |  |  |  |  |
| II | | 0.2495 | 1.7950 | 0.6726-4.6030 |  | 0.2070 | 1.9830 | 0.6847-5.7430 |
| III | | 0.0392 | 3.1920 | 1.2379-8.2330 |  | 0.0083 | 4.1659 | 1.4441-12.0180 |
| IV | | <0.0001 | 8.4200 | 3.2705-21.6790 |  | <0.0001 | 11.0267 | 3.8061-31.9450 |
| Histologicaltype | |  |  |  |  |  |  |  |
| Colon Adenocarcinoma | |  |  |  |  |  |  |  |
| Colon Mucinous Adenocarcinoma | | 0.3370 | 1.3216 | 0.7483-2.3340 |  | 0.4028 | 1.3026 | 0.7012-2.4200 |
| Discrepancy | | 0.9470 | 0.9347 | 0.1283-6.8080 |  | 0.6129 | 0.5897 | 0.0762-4.5620 |
| TMB | |  |  |  |  |  |  |  |
| low vs high | | 0.561 | 0.8845 | 0.5847-1.3380 |  | 0.1156 | 0.6898 | 0.4344-1.0960 |
| Risk score | |  |  |  |  |  |  |  |
| Increasing | | 0.0163 | 1.5270 | 1.0210-2.2820 |  | 0.0095 | 1.7318 | 1.2511-2.4220 |
| IGBRS, immune gene-set based recurrence signature; HR, hazard ratio. CI, confidence interval | | | | | | | | |

**Supplementary Figures**

**
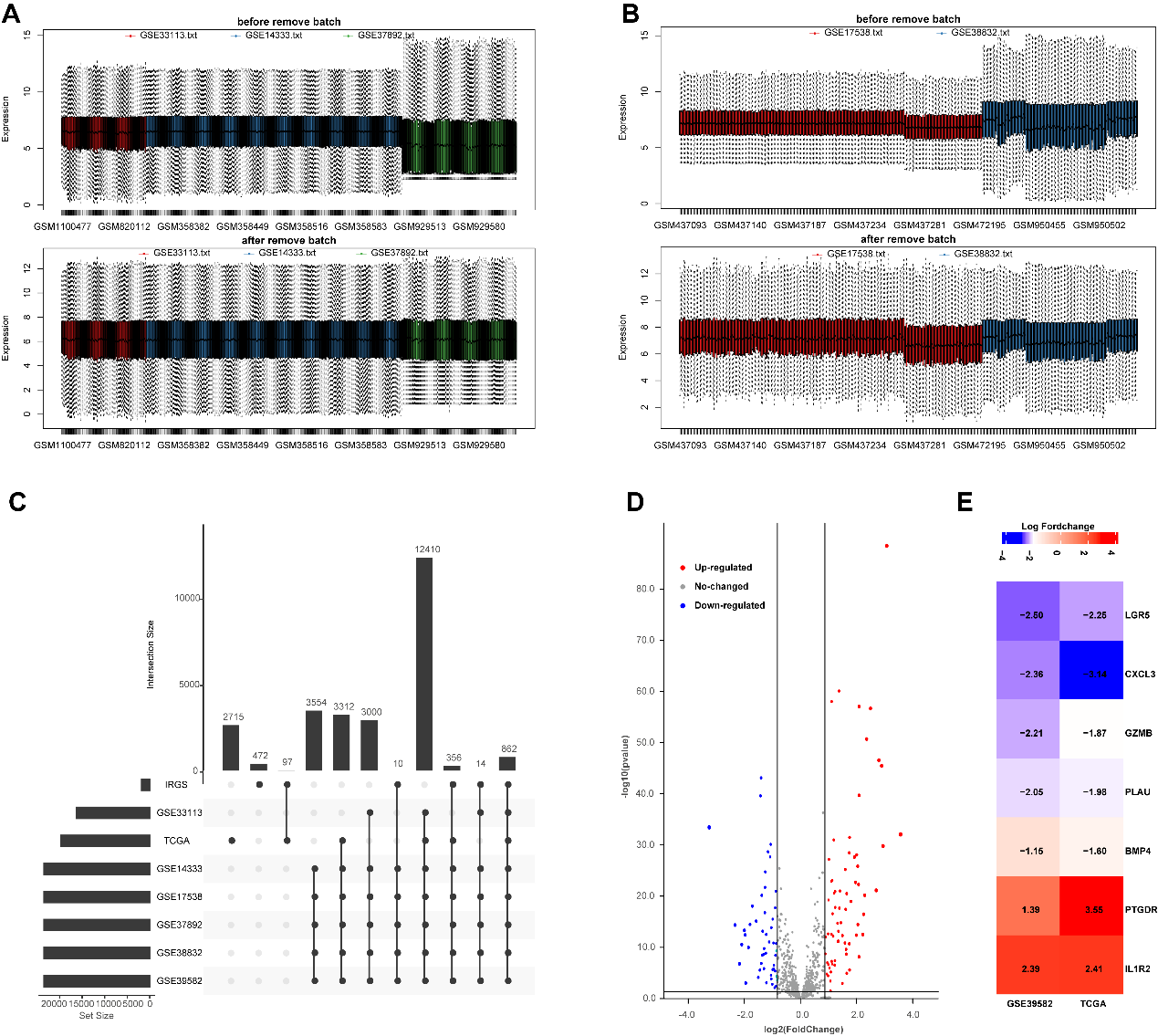
**

**Figure S1.****Data processing and screening differences IRGS between colorectal adenocarcinoma and para-carcinoma tissues**.**A.** The boxplot shows that we have merged the three datasets GSE14333, GSE37892, and GSE33113 and removed the batch effect. **B**. the merge of GSE17538 and GSE38832.Then, as the Upset plot shows, IRGS coincident in the TCGA, GSE14333, GSE37892, GSE33113, GSE17538, GSE38832, GSE39582, and ImmPort databases were screened (**C).** the difference analysis was performed among the colorectal adenocarcinoma and normal specimens in GSE39582 and TCGA cohorts (**D**). Red indicates high expression and blue indicates low expression. **E.** The log Fordchange value of 7 IRGs in GSE39582 and TCGA cohorts.


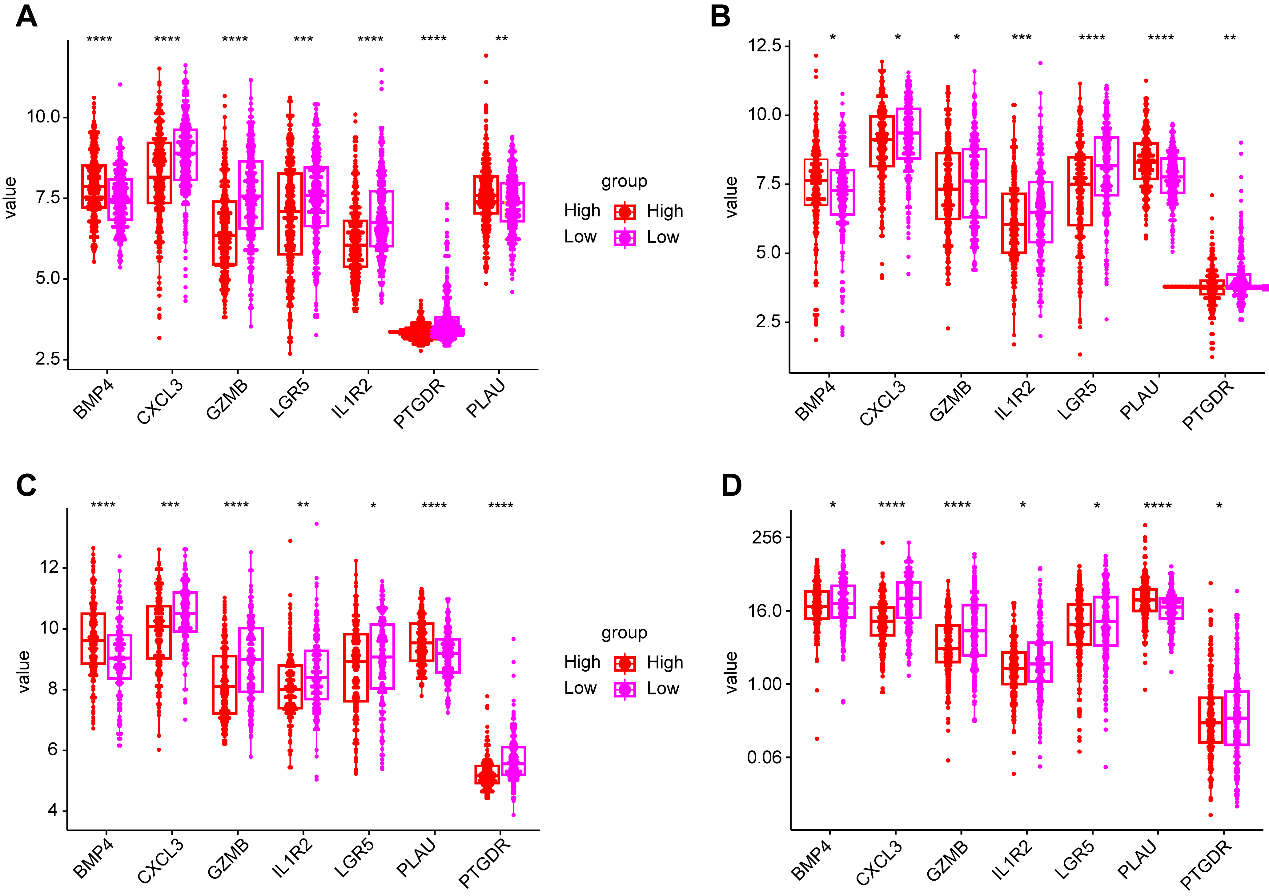


**Figure S2.** **Different expression of immune genes in high- and low-risk groups in training, test, validation, and TCGA cohorts. A.**trainingcohort; **B.** validation cohort.**C**. test cohort; **D**. TCGA cohort. *, **, *** and **** represent p < 0.05, p < 0.01, p < 0.001 and p < 0.0001, respectively.

**
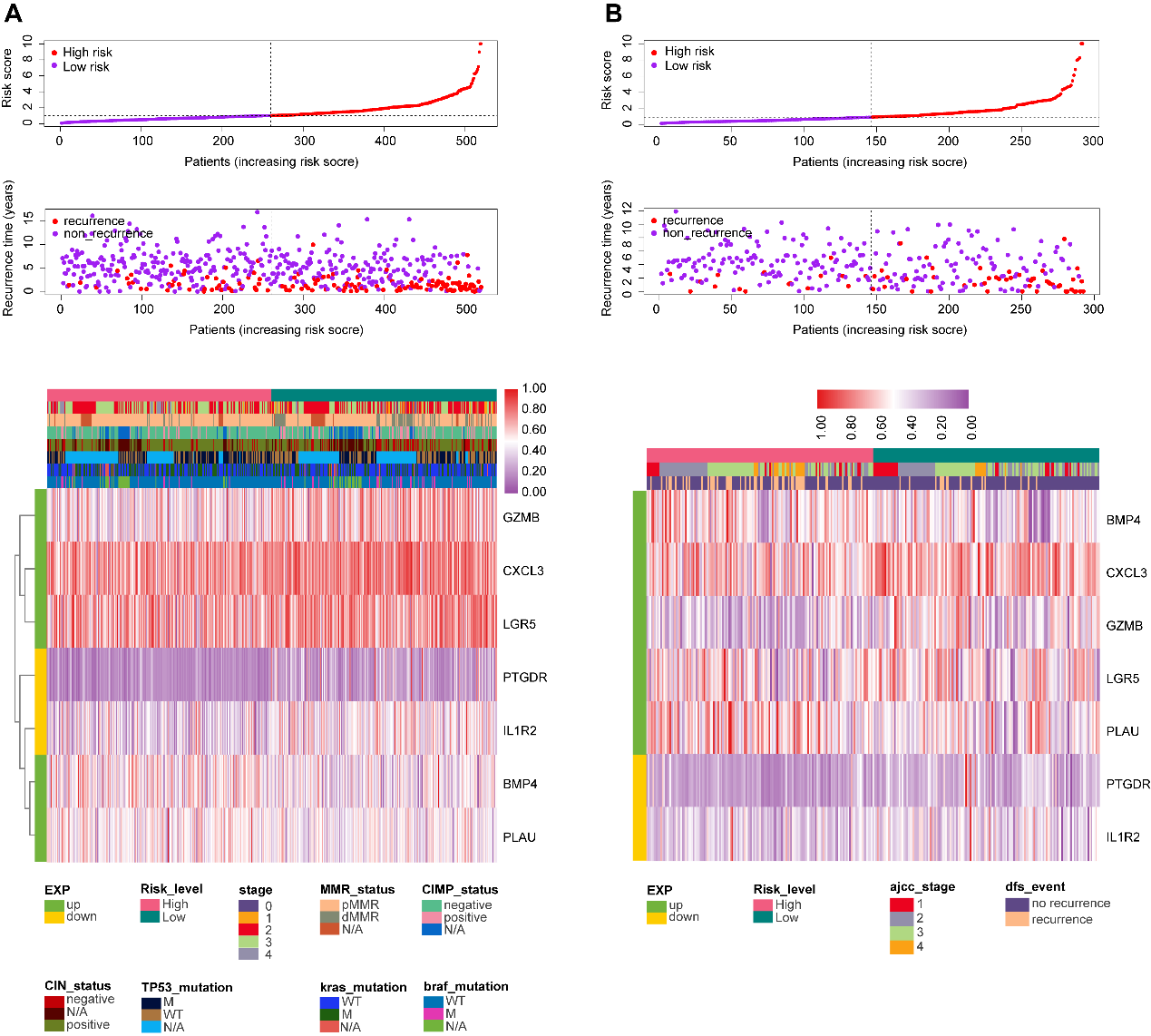
**

**Figure S3.****The distribution of risk score, recurrence status, and gene expression panel.** Correlation between IGBRS and the RFS of patients in the training (**A**) and test (**B**) cohort. The distribution of risk scores (upper), recurrence time (middle), and IRGS expression levels (lower). The black dotted lines represent the median risk score cut-off dividing patients into low- and high-risk score groups. The red dots and lines represent the patients in the high-score groups. The green dots and lines represent the patients in the low-score groups.

**
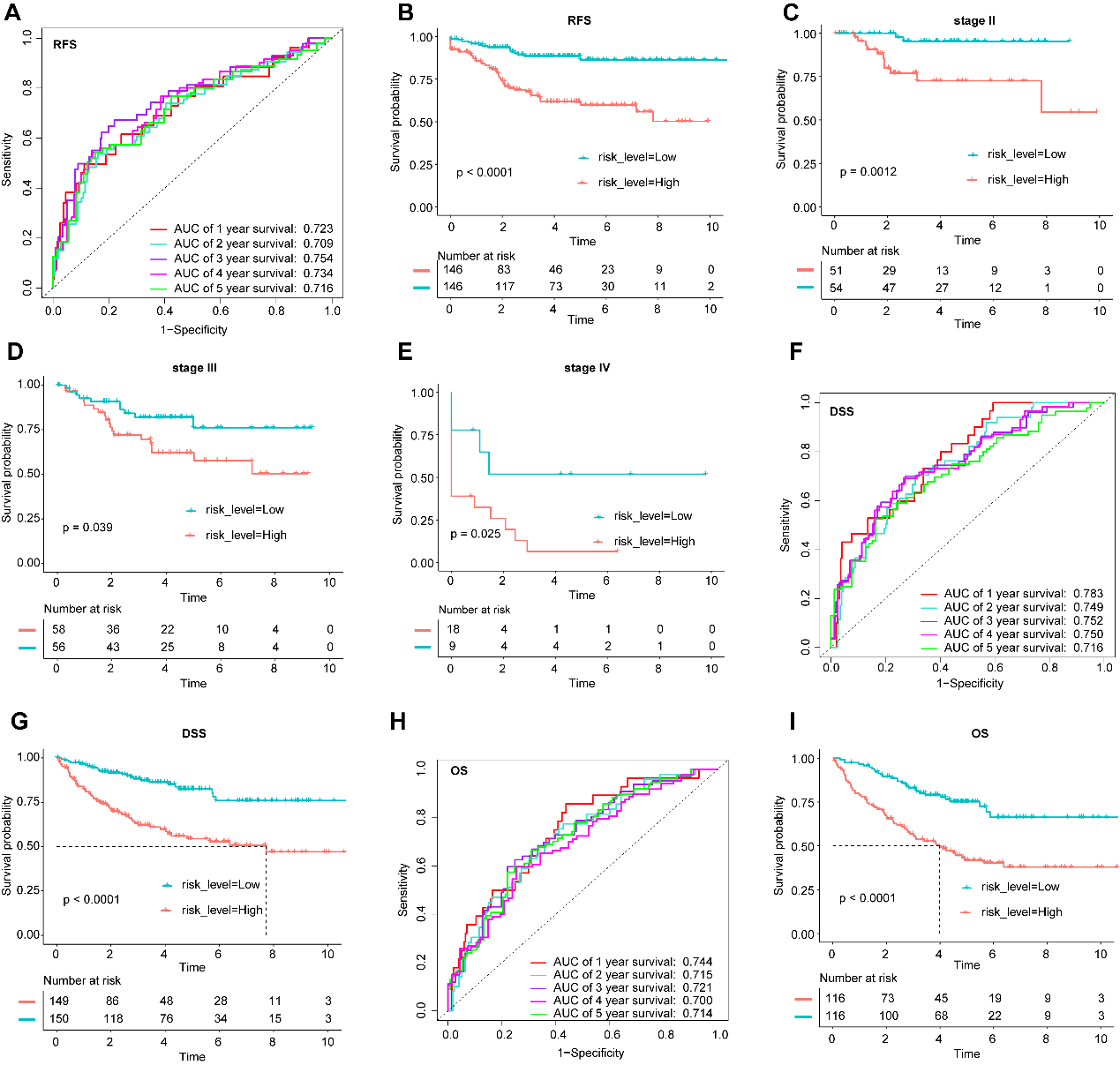
**

**Figure S4. Application of IGBRS in predicting RFS, DSS, and OS of CRC in test cohort**. **A.**ROC analysis of immune related gene signature for prediction of RFS at 1, 2, 3, 4, and 5 years in test cohort; **B.** Kaplan-Meier curves of RFS in all CRC patients of test cohort based on risk score; **C.**Kaplan-Meier curves of RFS in stage II CRC patients of test cohort based on risk score; **D.** Kaplan-Meier curves of RFS in stage III CRC patients of test cohort based on risk score; **E.** Kaplan-Meier curves of RFS in stage IV CRC patients of test cohort based on risk score; **F.** ROC analysis of immune related gene signature for prediction of DSS at 1, 2, 3, 4, and 5 years in test cohort; **G.** Kaplan-Meier curves of DSS in all CRC patients of test cohort based on risk score. **H.** ROC analysis of immune related gene signature for prediction of OS at 1, 2, 3, 4, and 5 years in test cohort; **I.** Kaplan-Meier curves of OS in all CRC patients of test cohort based on risk score.

**
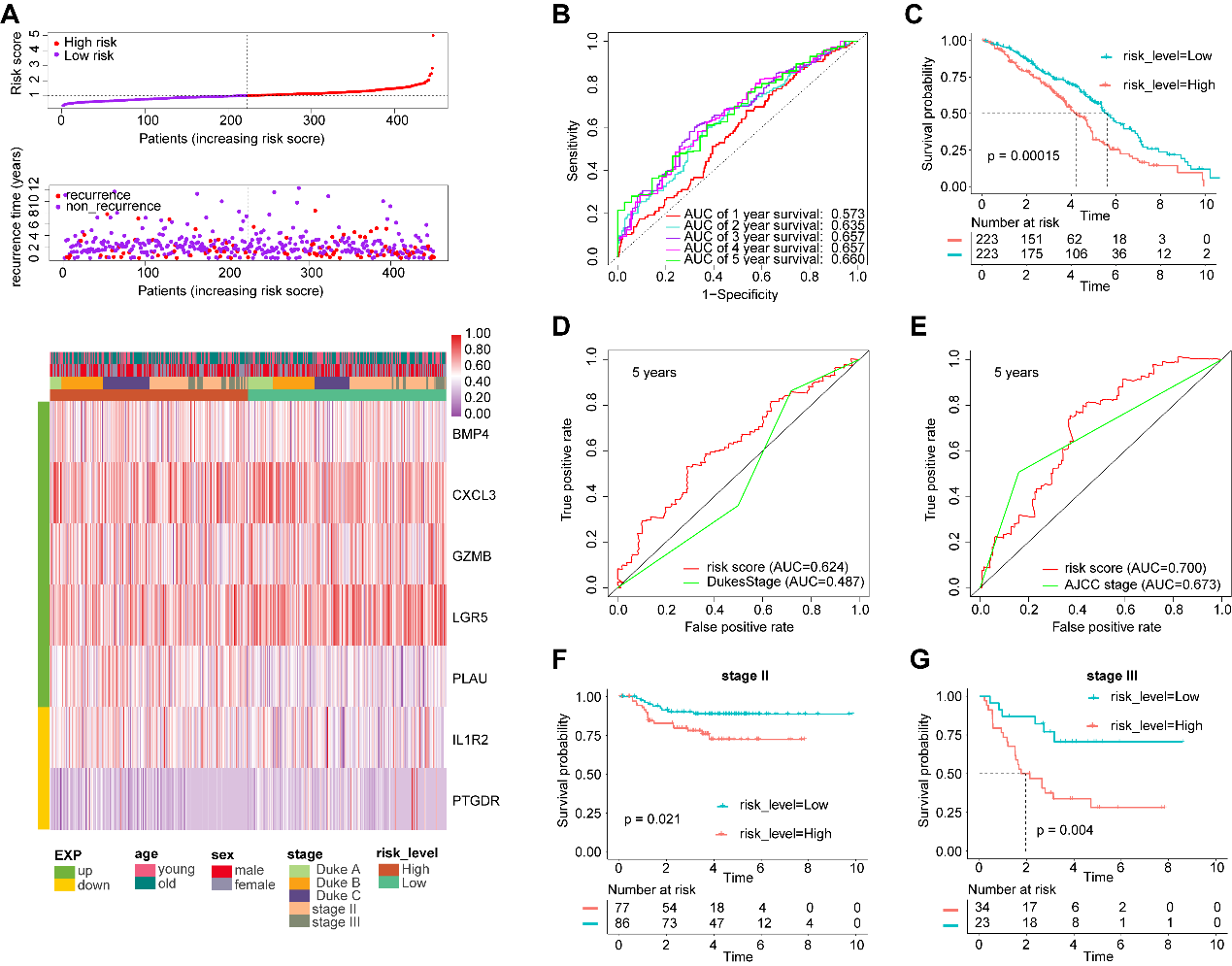
**

**Figure S5. Application of IGBRS in predicting RFS of CRC in validation cohort**. **A**. The distribution of risk score, recurrence status, and gene expression panel in validation cohort.**B**. ROC analysis of immune related gene signature for prediction of RFS at 1, 2, 3, 4, and 5 years in validationcohort;**C.** Kaplan-Meier curves of RFS in all CRC patients of validation cohort based on risk score;**D.** ROC analysis of immune related gene signature and Duke stage for prediction of RFS at 5 years in GSE17538 cohort;**E.** ROC analysis of immune related gene signature and TNM stage for prediction of RFS at 5 years in validation cohort;**F.** Kaplan-Meier curves of RFS in stage II CRC patients of validation cohort based on risk score; **G.**Kaplan-Meier curves of RFS in stage III CRC patients of validation cohort based on risk score.

**
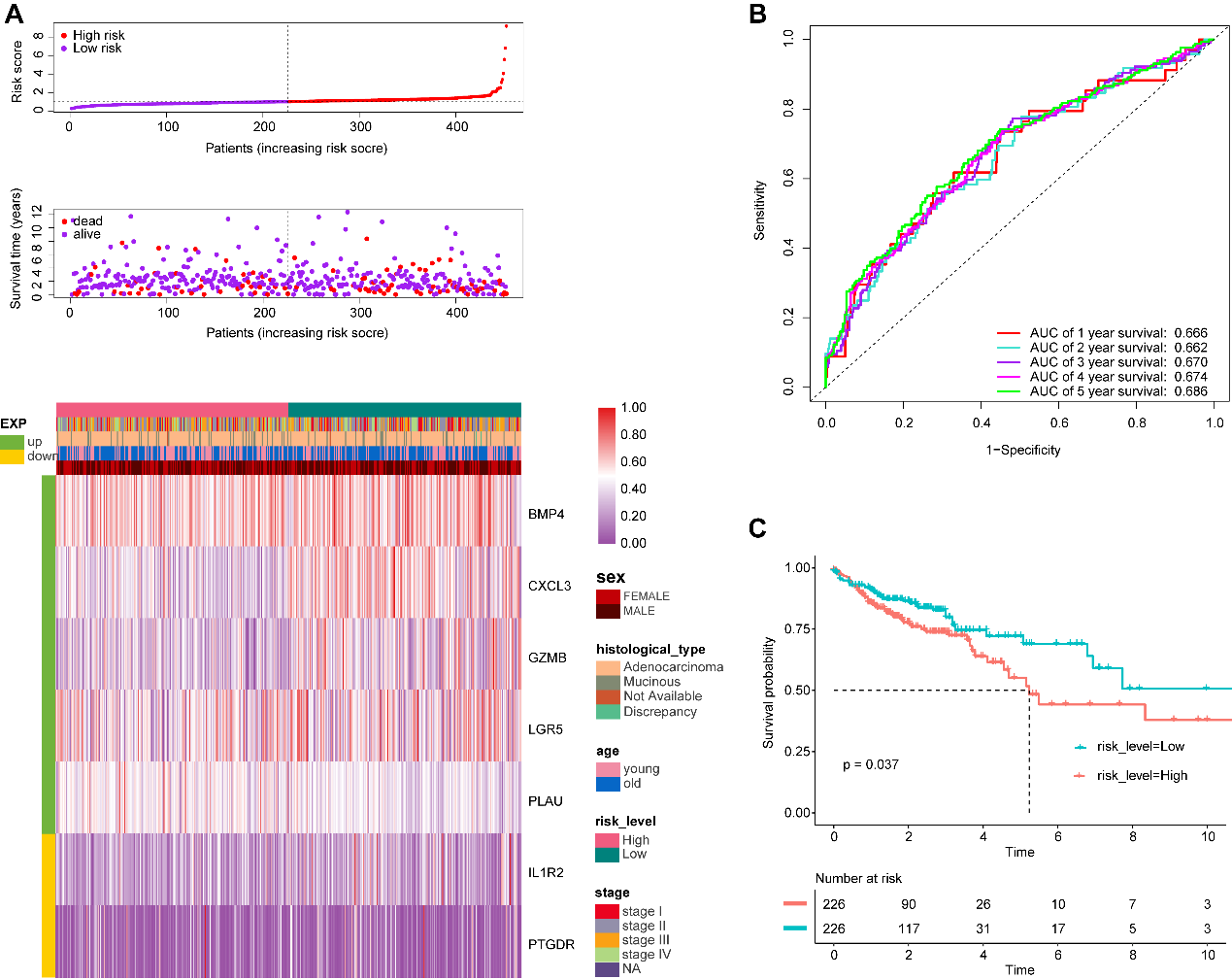
**

**Figure S6. Application of IGBRS in predicting OS of CRC in TCGA cohort**.**A**. The distribution of risk score, recurrence status, and gene expression panel in TCGA cohort.**B**. ROC analysis of immune related gene signature for prediction of OS at 1, 2, 3, 4, and 5 years in TCGAcohort;**C.** Kaplan-Meier curves of OS in all CRC patients of TCGA cohort based on risk score.

**
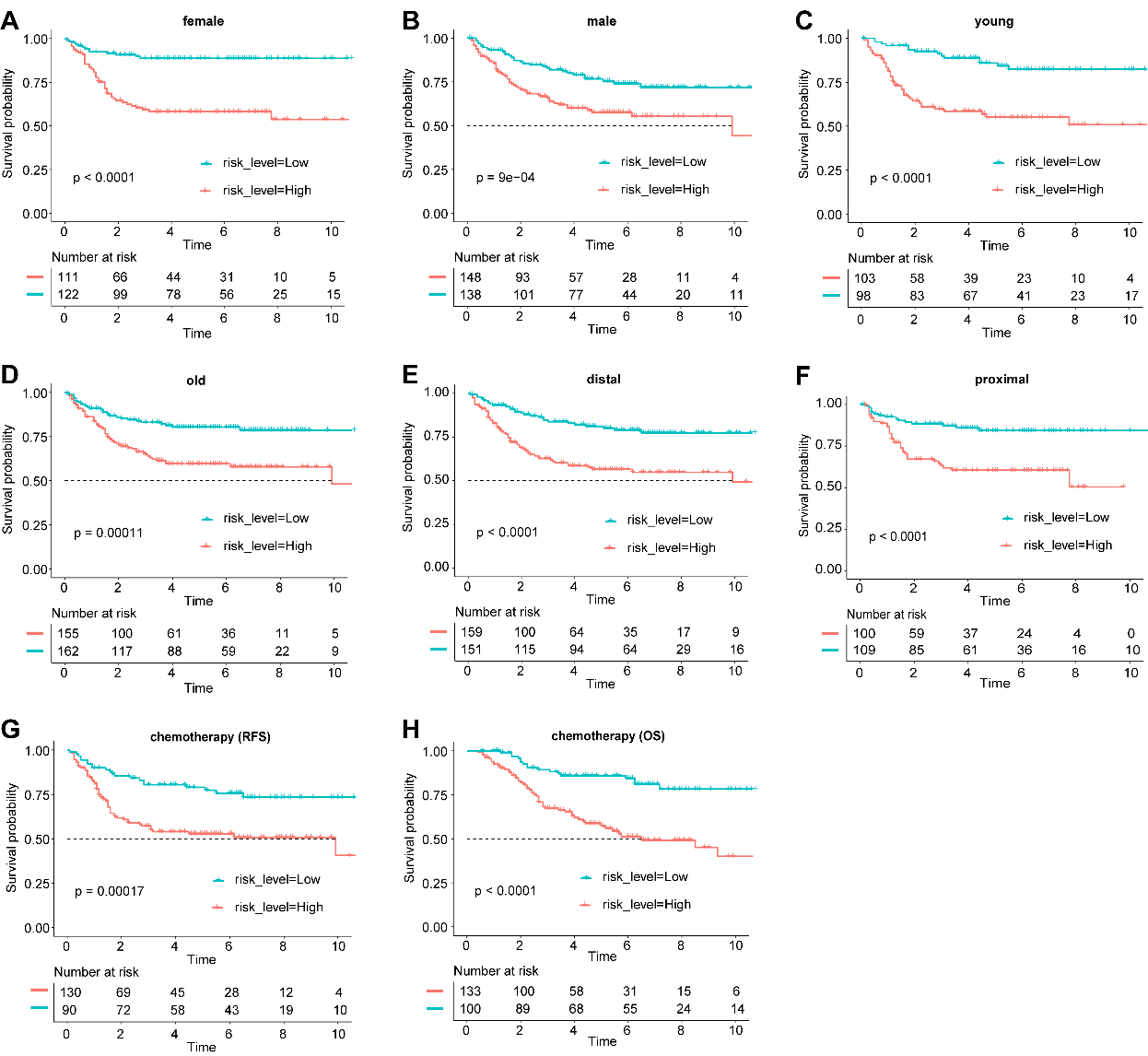
**

**Figure S7. Kaplan-Meier curves of RFS in the different clinical subtype of CRC based on risk score in the training cohort, respectively. Aand B.** Kaplan-Meier curves of RFS in different gender of CRC based on risk score, respectively**. C and D.** Kaplan*-*Meier curves of RFS in different age of CRC based on risk score, respectively. **E and F.** Kaplan*-*Meier curves of RFS in different location of CRC based on risk score, respectively; **G** and **H.** Kaplan-Meier curves of RFS and OS in CRC patients with chemotherapy based on risk score, respectively.

**
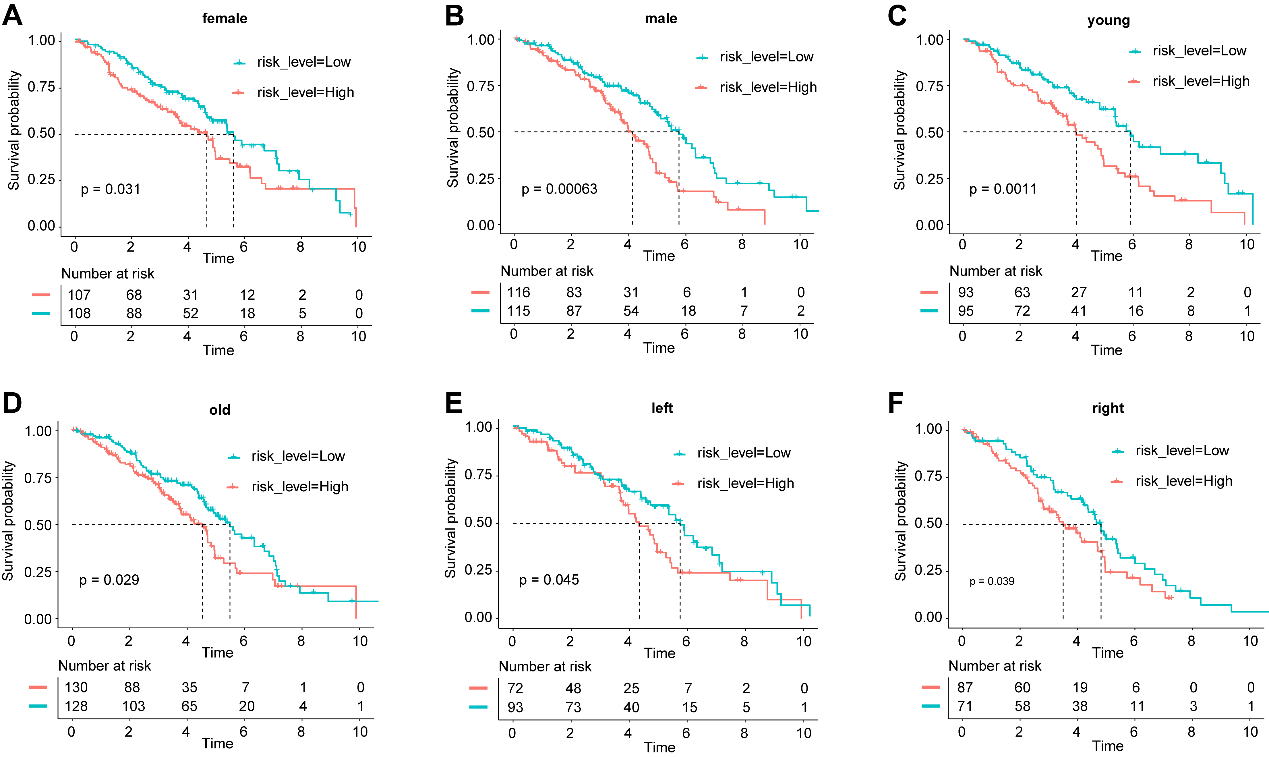
**

**Figure S8. Kaplan-Meier curves of RFS in the different clinical features of CRC based on risk score in the validation cohort, respectively. Aand B.** Kaplan-Meier curves of RFS in different gender of CRC based on risk score, respectively**. C and D.** Kaplan*-*Meier curves of RFS in different age of CRC based on risk score, respectively. **E and F.** Kaplan*-*Meier curves of RFS in different location of CRC based on risk score, respectively.

**
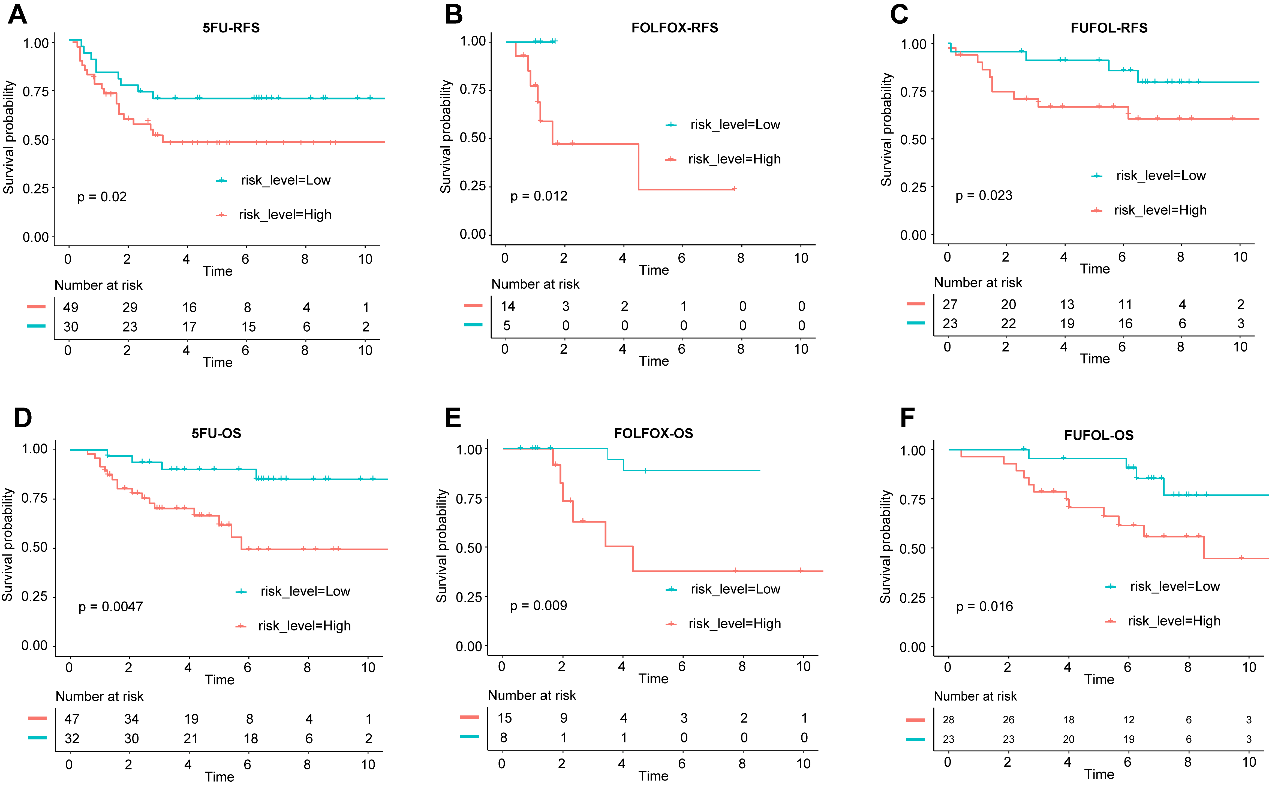
**

**Figure S9. Kaplan-Meier curves of RFS and OS in the different subtype of chemotherapeutic based on risk score in the training cohort, respectively.**

**
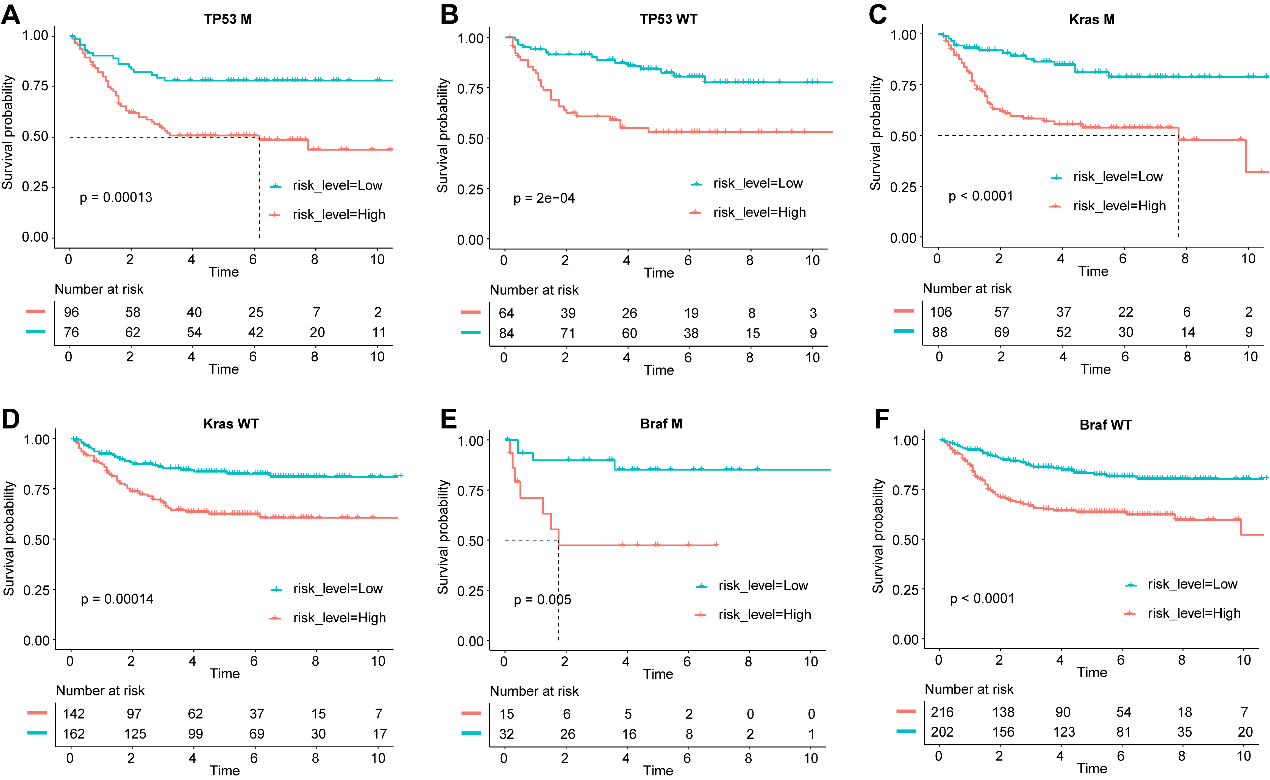
**

**Figure S10.IGBRS can predict RFS independent of TP53, KRAS or BRAF mutation status.** Kaplan-Meier curves of RFS in the different subtype of TP53 (**A** and **B**), KRAS (**C** and **D**), and BRAF (**E** and **F).**


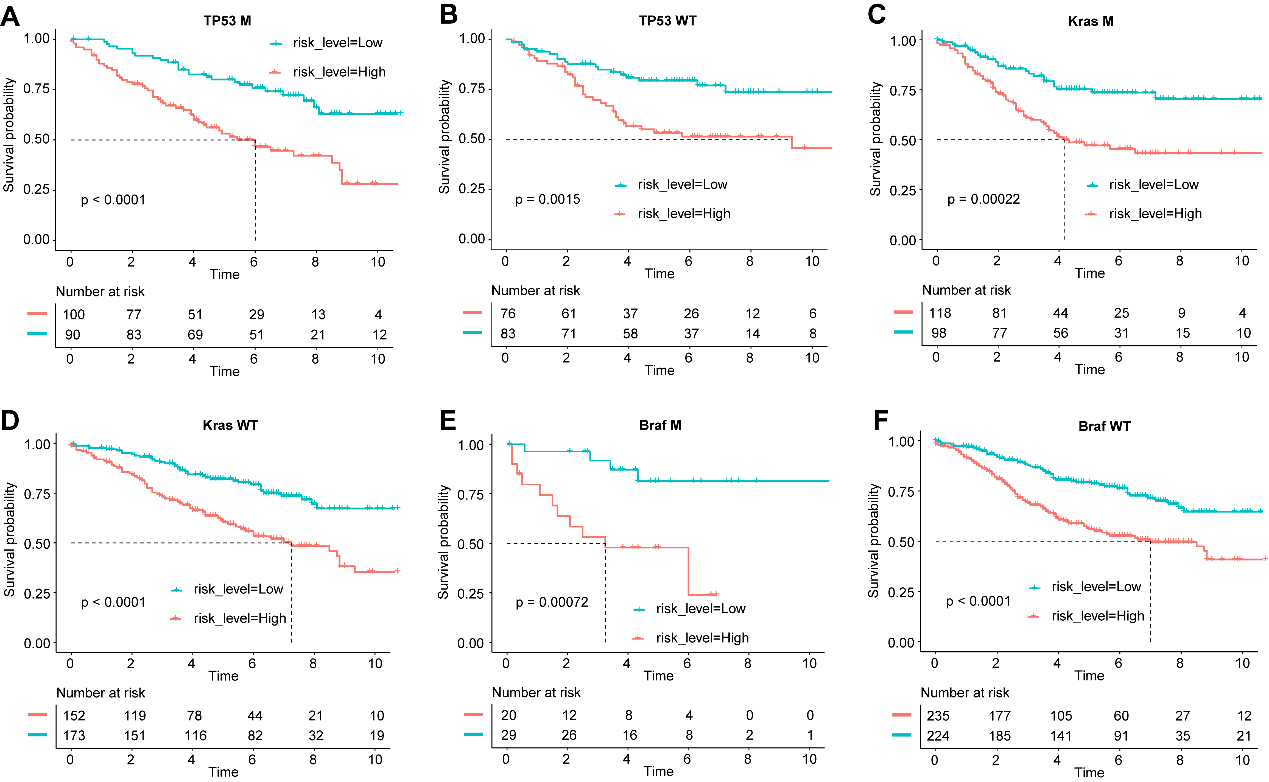


**Figure S11.IGBRS can predict OS independent of TP53, KRAS or BRAF mutation status.** Kaplan-Meier curves of OS in the different subtype of TP53 (**A** and **B**), KRAS (**C** and **D**), and BRAF (**E** and **F)**.


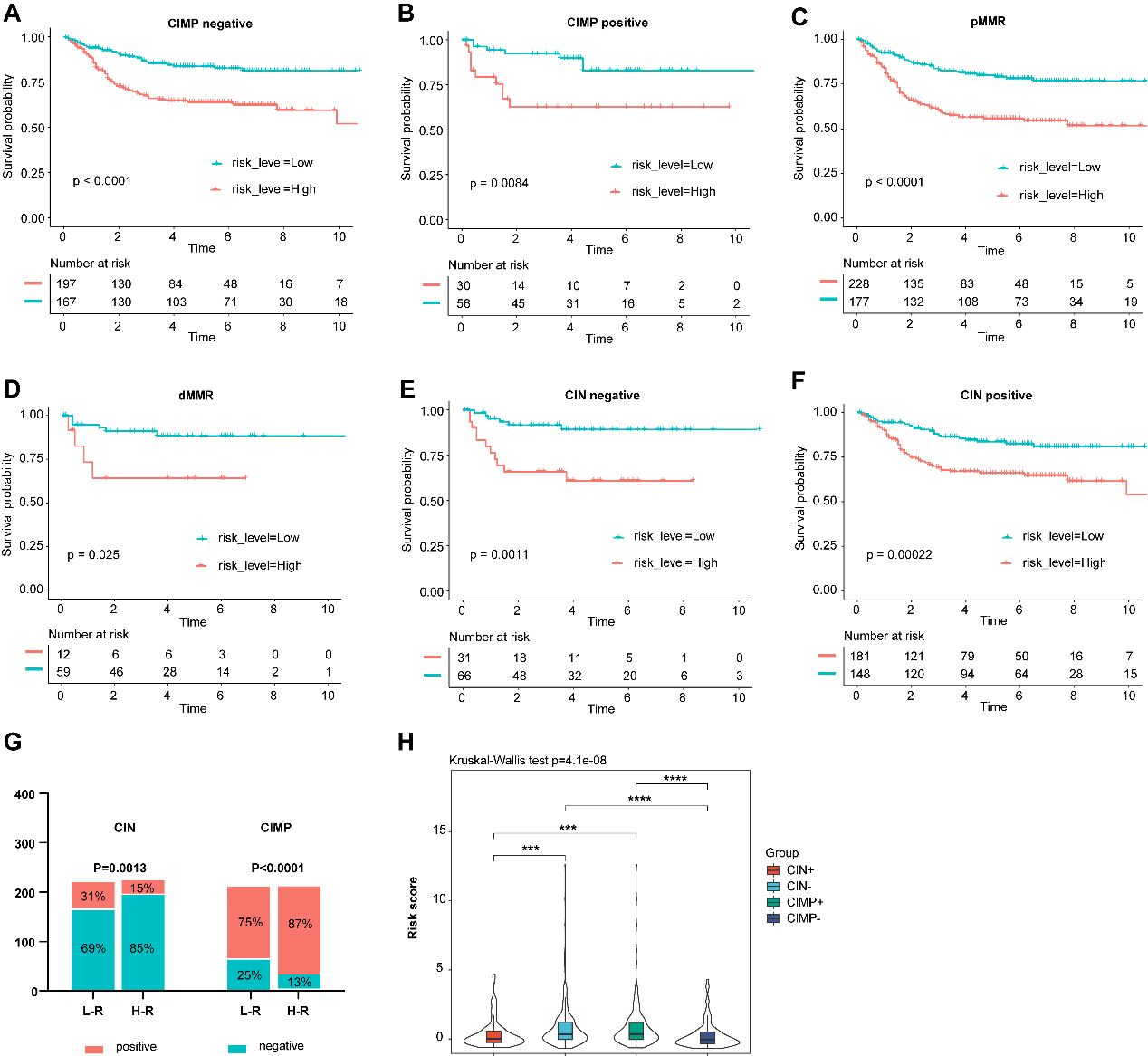


**Figure S12.****Kaplan-Meier curves of RFS in the different subtype of CIN, MMR, and CIMP status based on risk score in the training cohort, respectively.** Kaplan-Meier curves of RFS in the different subtype of CIMP (**A** and **B**), MMR (**C** and **D**), and CIN (**E** and **F)**; **G.**Proportion of negative and positive of CIN and CIMP in the high-risk (H-R) and low-risk (L-R) groups; **H**.Differences between negative and positive of CIN and CIMP.*, **, *** and **** represent p < 0.05, p < 0.01, p < 0.001 and p < 0.0001, respectively.


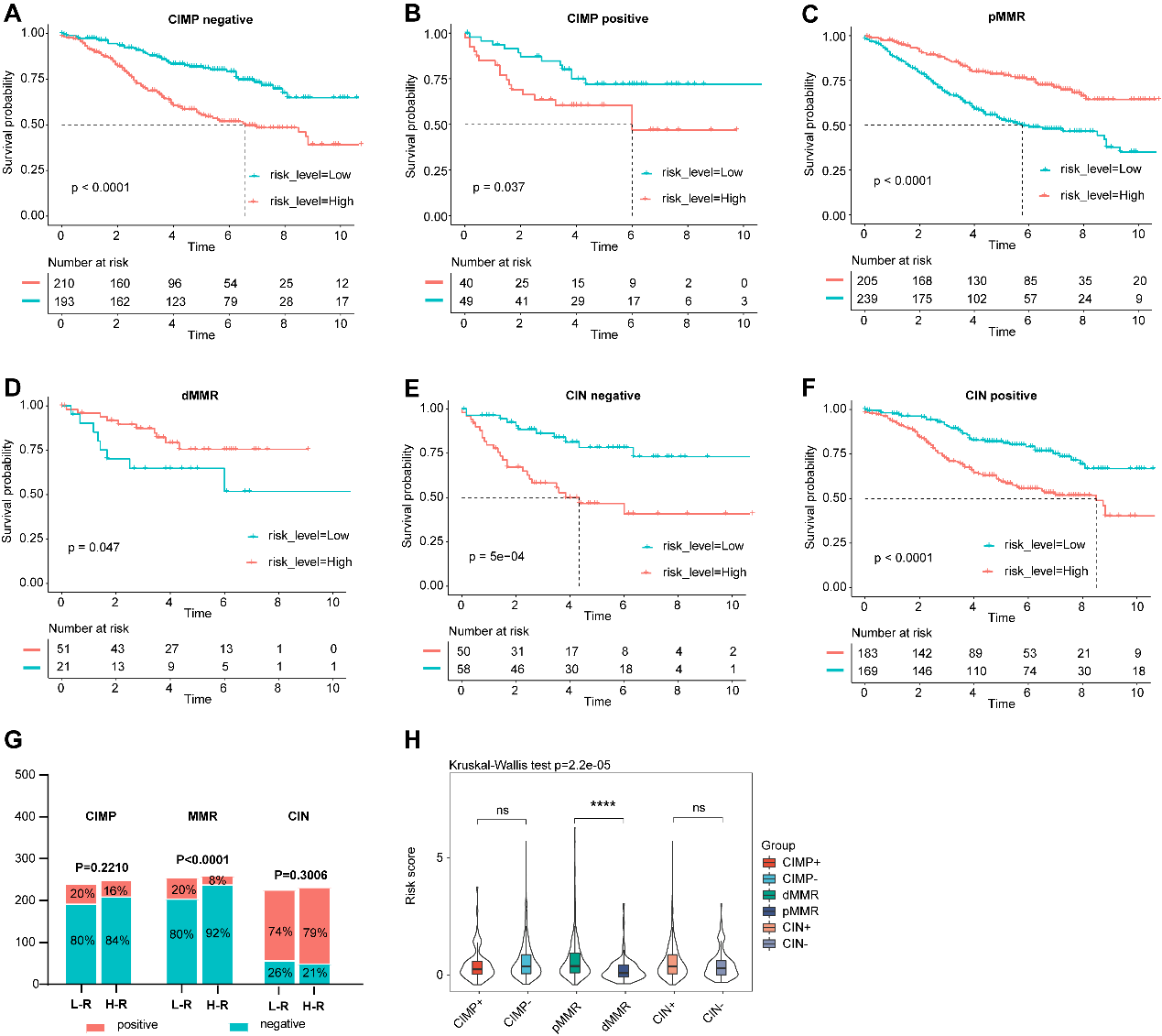


**Figure S13.Kaplan-Meier curves of OS in the different subtype of CIN, MMR, and CIMP status based on risk score in the training cohort, respectively.** Kaplan-Meier curves of RFS in the different subtype of CIMP (**A** and **B**), MMR (**C** and **D**), and CIN (**E** and **F)**; **G.**Proportion of negative and positive of CIN, MMR, and CIMP in the high-risk (H-R) and low-risk (L-R) groups; **H**.Differences between negative and positive of CIN, MMR, and CIMP.*, **, *** and **** represent p < 0.05, p < 0.01, p < 0.001 and p < 0.0001, respectively.

**
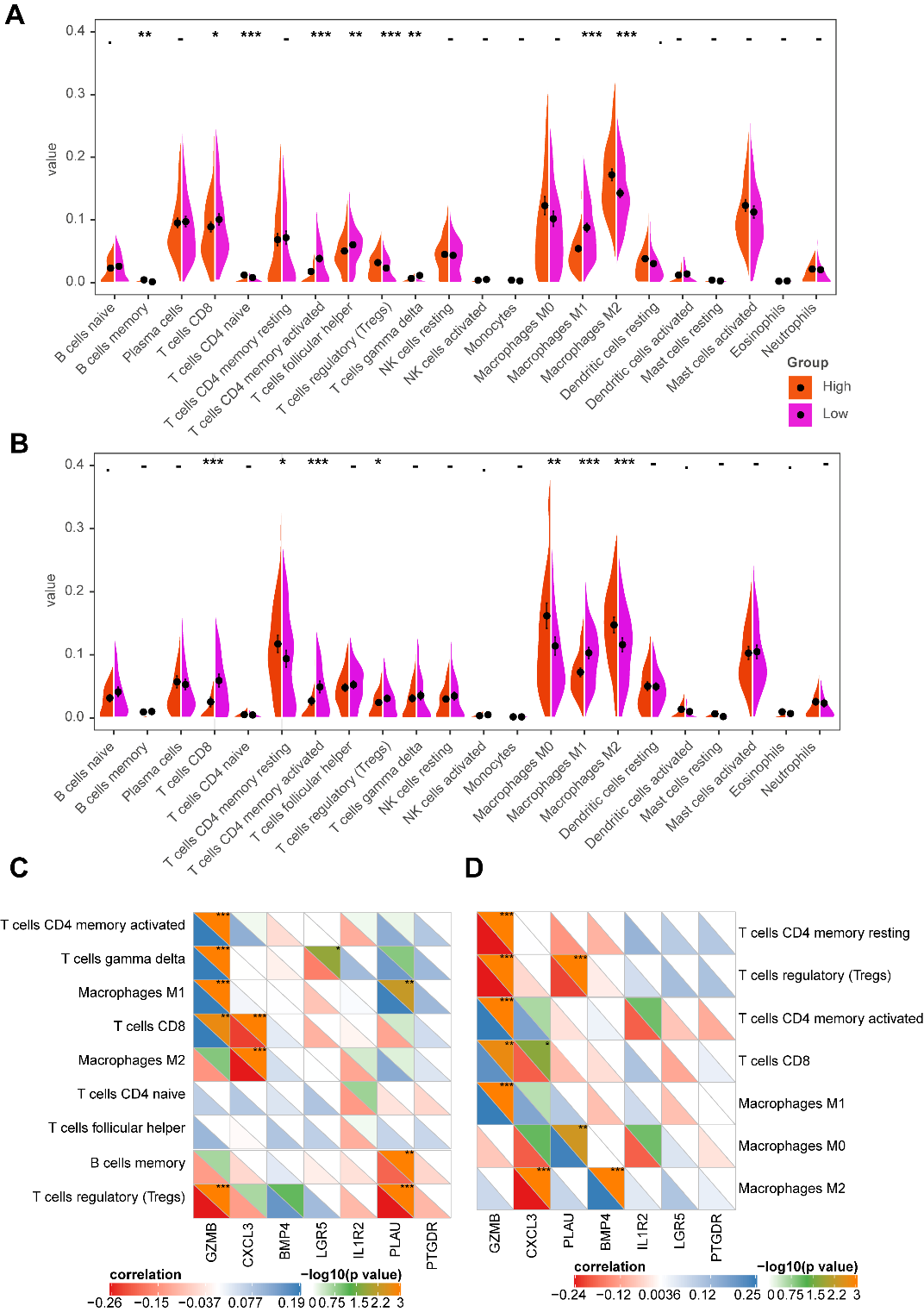
**

**Figure S14.Landscape of immune** **infiltration cells** **in the training and test cohorts. A and B.** Different infiltration abundance of immune infiltration cells in high- and low-risk groups in the training(**A**) and test (**B**) cohorts, respectively.**C and D.**the Pearson correlation coefficients of 7 IRGS with various differential expressed immune infiltration cells in the training(**C**) and test (**D**) cohorts.*, **, *** and **** represent FDR< 0.05, FDR< 0.01, FDR< 0.001 and FDR< 0.0001, respectively.


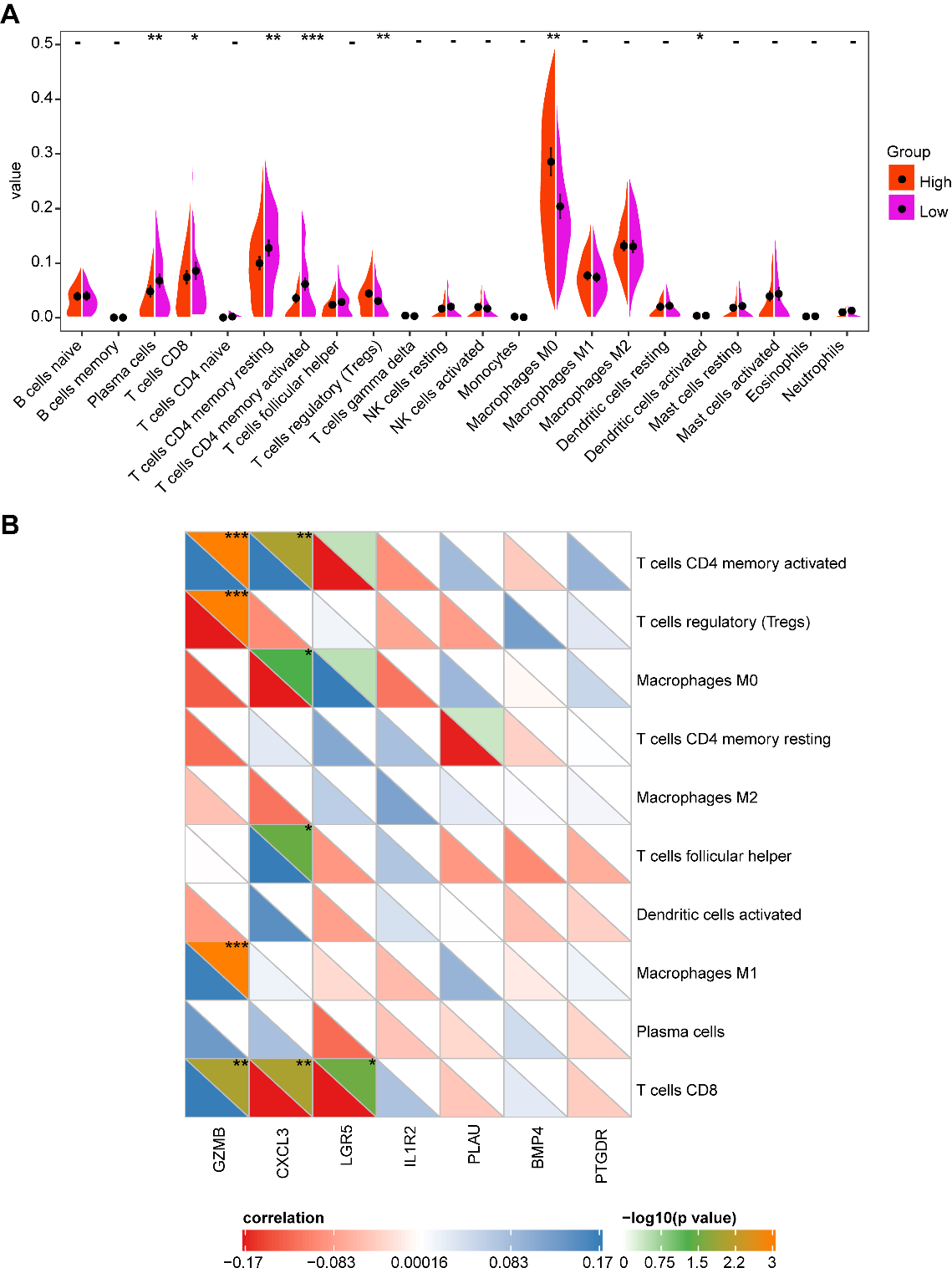


**Figure S15.****Landscape of immune infiltration cells in the TCGA cohort. A.** Different infiltration abundance of immune infiltration cells in high- and low-risk groups in TCGA cohorts.**B.**the Pearson correlation coefficients of 7 IRGS with various differential expressed immune infiltration cells in TCGAcohort.*, **, *** and **** represent FDR< 0.05, FDR< 0.01, FDR< 0.001 and FDR< 0.0001, respectively.


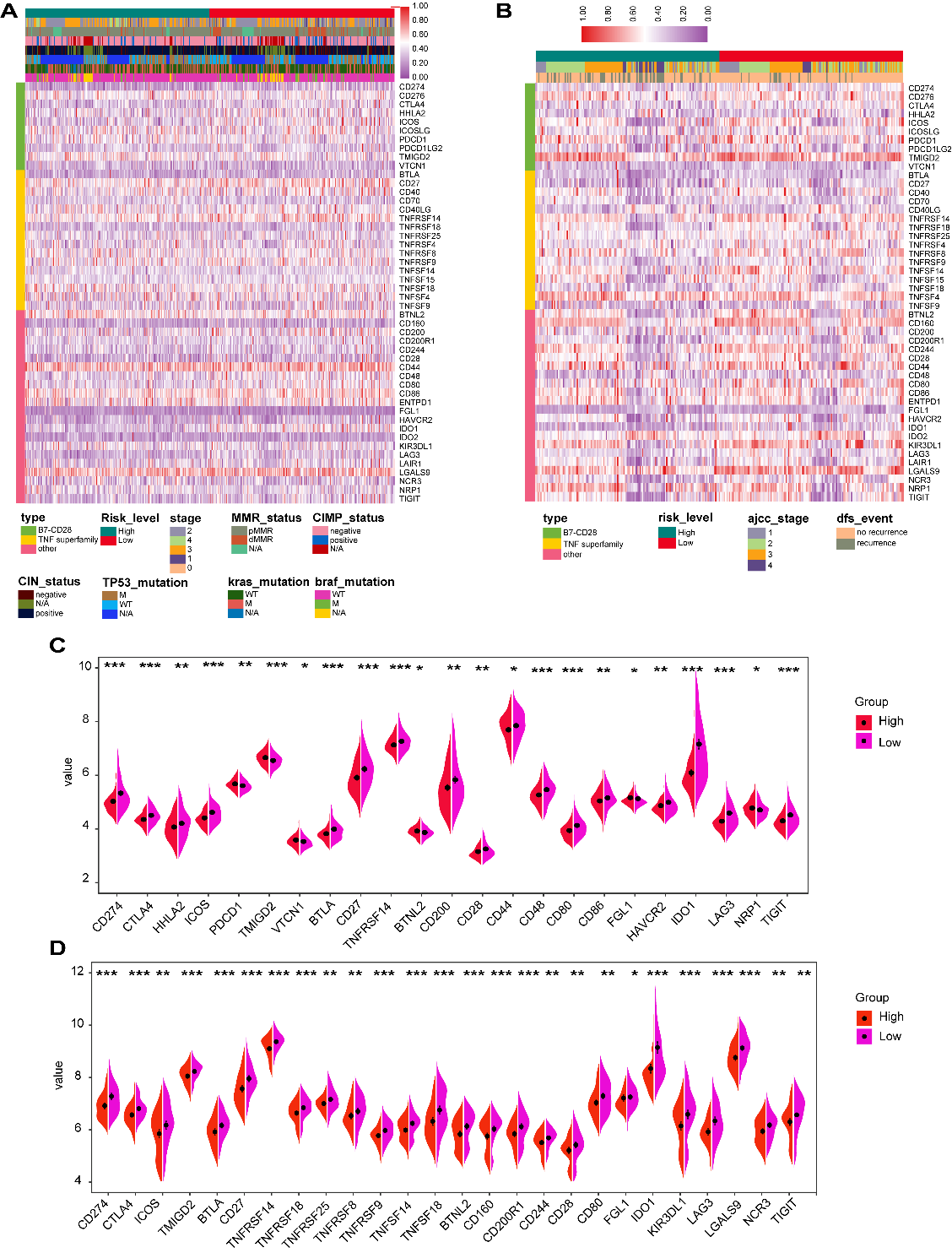


**Figure S16. The relationship between immune checkpoints and risk scores.A-B.** The expression profile of costimulatory/coinhibitory immune checkpoints landscape in training (**A**) and test (**B**) cohorts, respectively; **C-D.** Different expression of immune checkpoints in high- and low-risk groups in training (**C**) and test (**D**) cohorts, respectively.

**
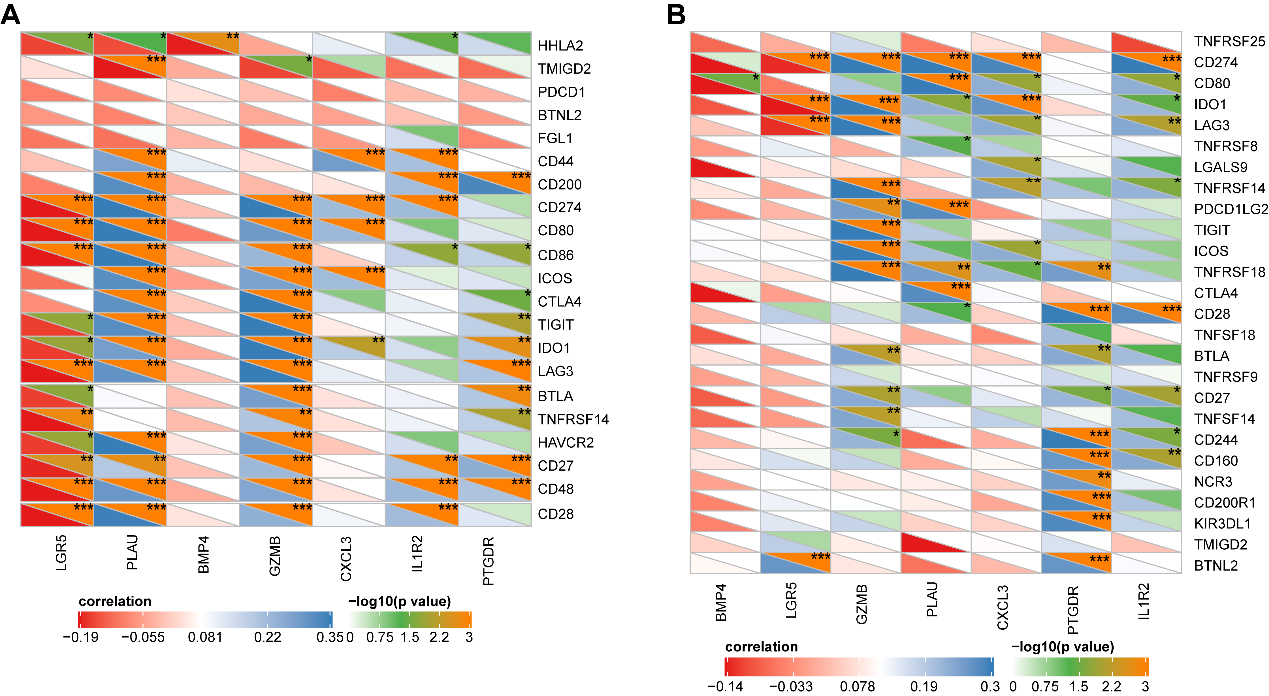
**

**Figure S17.****the Pearson correlation coefficients of 7 IRGS with various differential expressed immune checkpoints in the training and testcohorts.(A)** training cohort. **(B)** test cohort.*, **, *** and **** represent FDR< 0.05, FDR< 0.01, FDR< 0.001 and FDR< 0.0001, respectively.

**
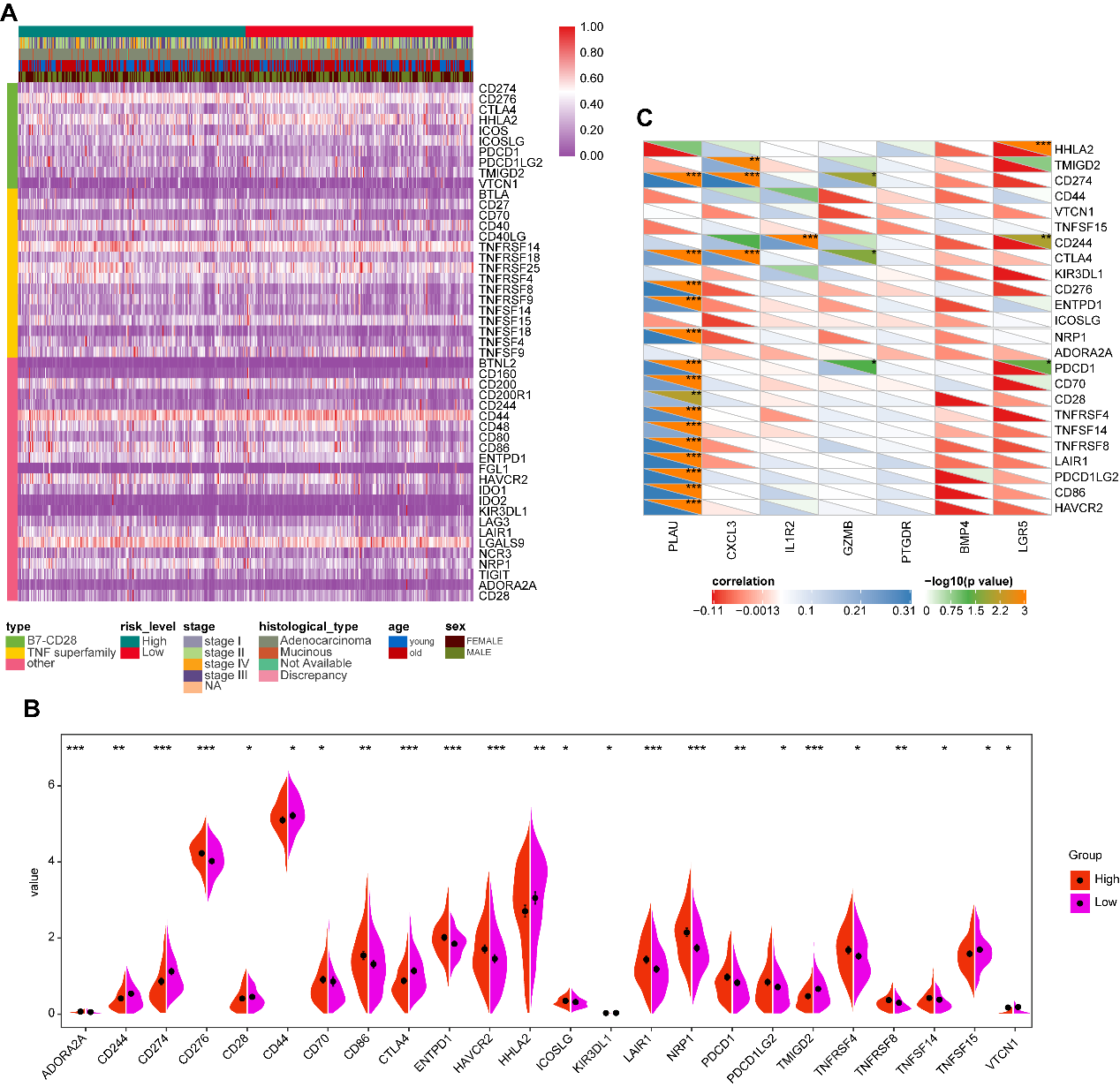
**

**Figure S18.****Landscape of immune infiltration checkpoints in the TCGA cohort.**

**A.** The expression abundance heatmap of 49 immune checkpoints**B**. Different expression abundance of immune checkpoints in high- and low-risk groups in TCGA cohorts. **C.** the Pearson correlation coefficients of 7 IRGS with various differential expressed immune checkpoints in TCGAcohort. *, **, *** and **** represent FDR< 0.05, FDR< 0.01, FDR< 0.001 and FDR< 0.0001, respectively.

**
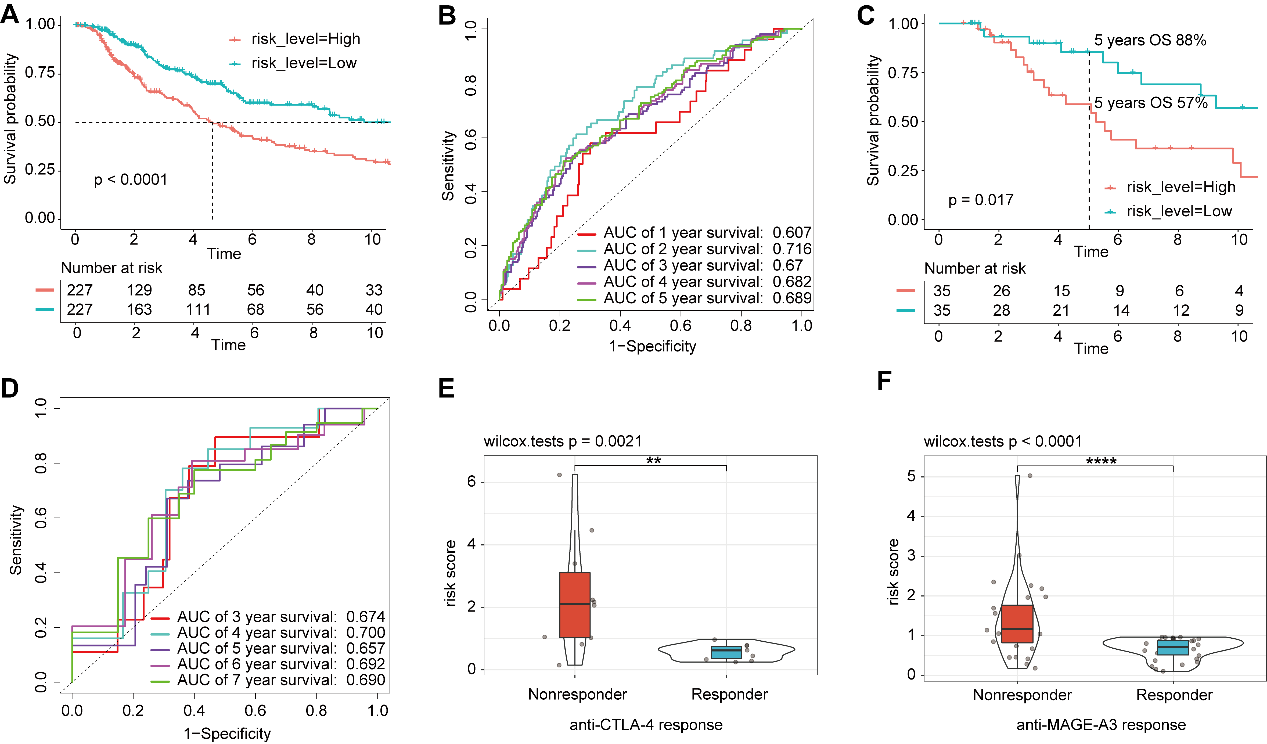
**

**Figure S19. Application of signature in immunotherapy. A.**Kaplan-Meier curves of OS in all cutaneous melanoma patients of TCGA-SKCMcohort based on risk score.**D.** ROC analysis of immune related gene signature for prediction of OS at 1, 2, 3, 4, and 5 years in TCGA-SKCM cohort.**C.**Kaplan–Meier curves of overall survival of patientstreated with immunotherapy in TCGA-SKCM cohort.**D.** Receiver operating characteristic (ROC) curves forsignature in the TCGA-SKCM cohort.**E.** Risk score in patients with response (blue) versusthose without response(red) (Wilcoxon P = 0.0021) toanti-CTLA-4 treatment.**F.** Risk score in patients with response (blue) versusthose without response(red) (Wilcoxon P <0.0001) toanti-MAGE-A3 treatment.

**
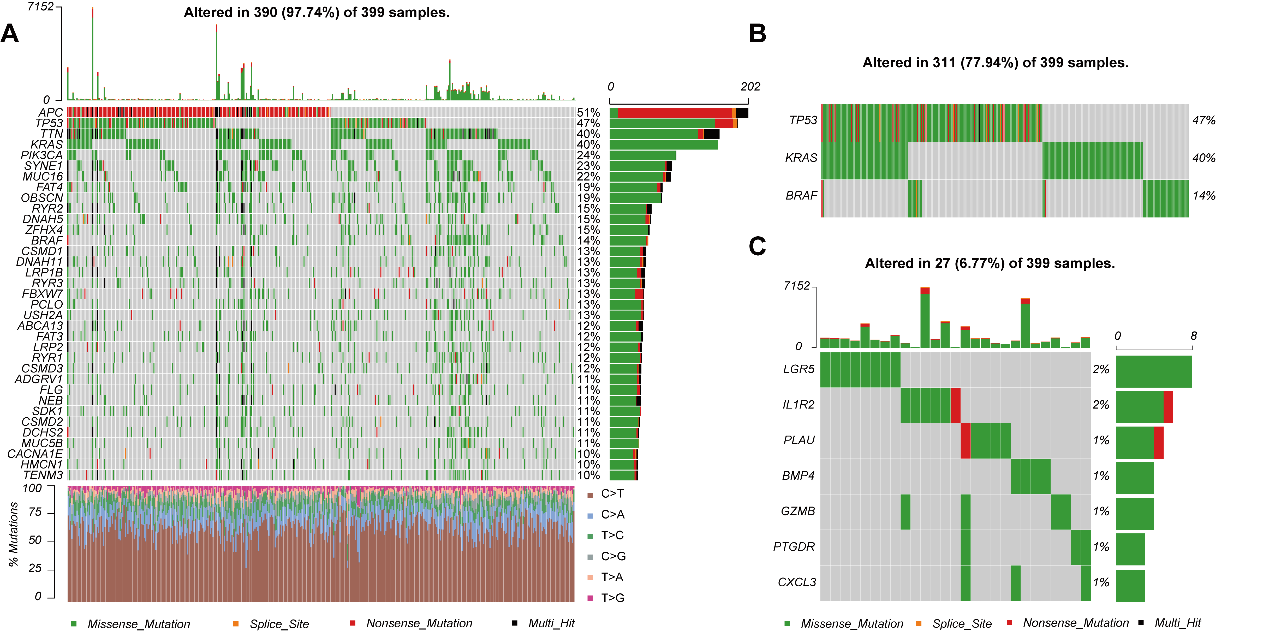
**

**Figure S20. Tumor somatic mutation landscape of the IGBRS.A.** the top 30 of gene mutations in the TCGA-COAD cohort. **B**. The mutations of the commonly mutant genes TP53, BRAF, and KRAS in the TCGA-COAD cohort. **C**. the mutation landscape of the 7 IGRs in IGBRS.

**
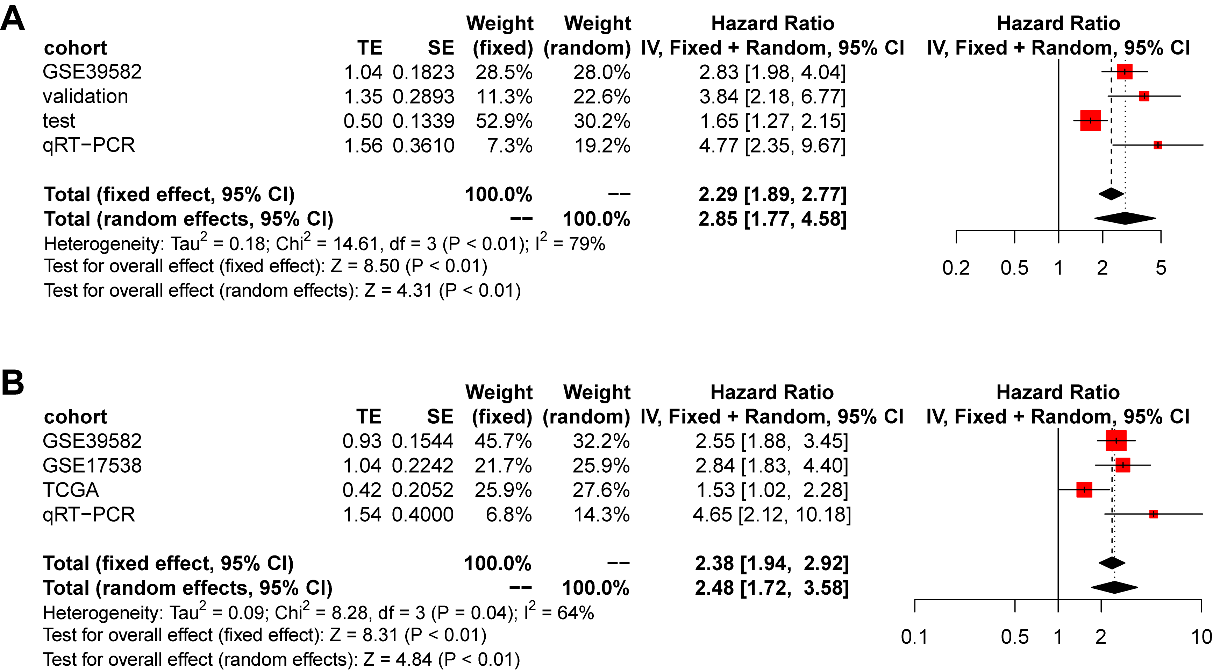
**

**Figure S21.** **Prognostic meta-analysis among training, test, validation, and TCGA cohorts.**


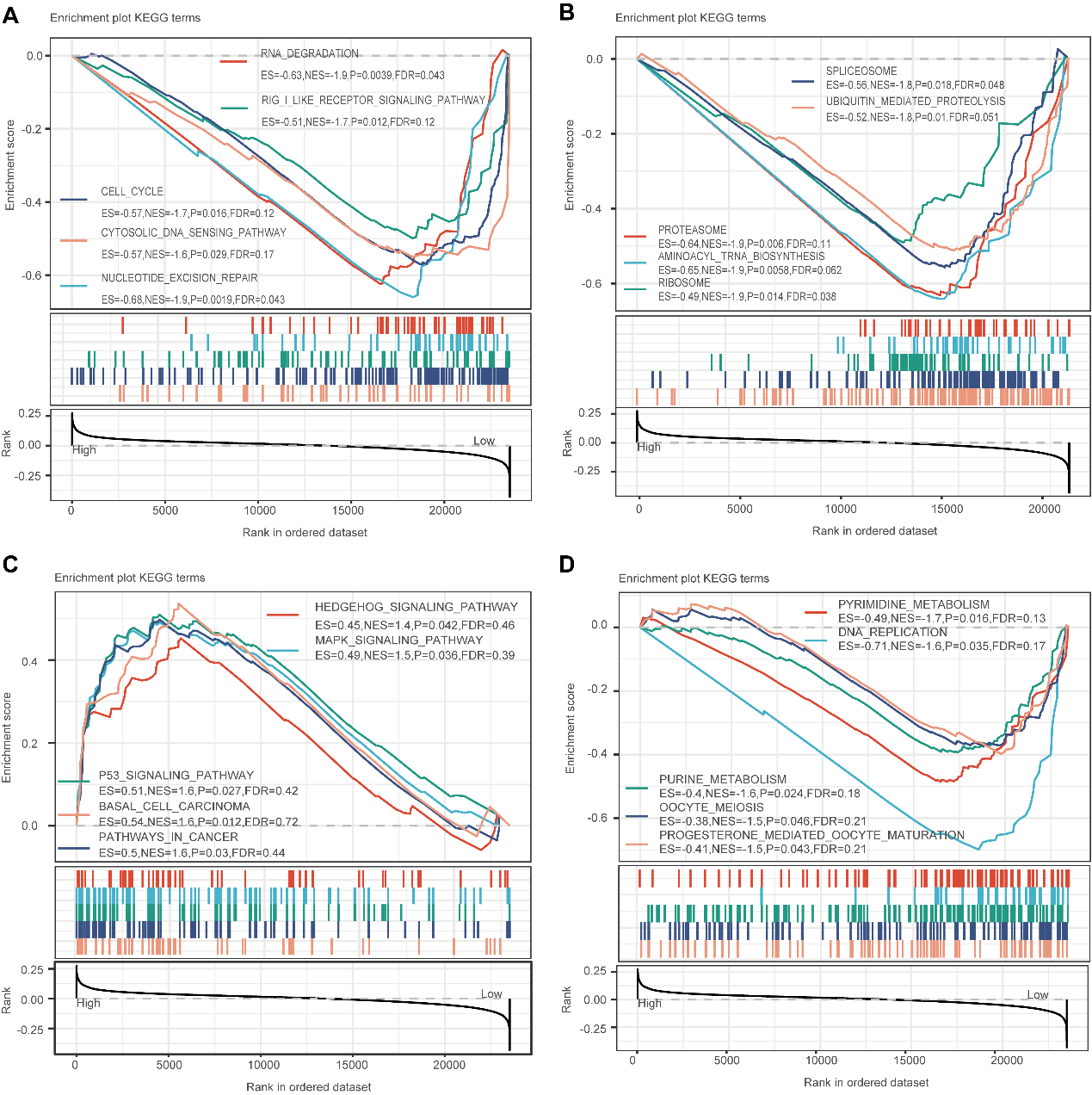


**Figure S22.** **GSEA analysis.** ES, enrichment score; NES, normalized enrichment score; P, P-value; FDR, adjusted P-value.
